# Unveiling Molecular Basis of *Terminalia chebula* in Modulating the AGE-RAGE-PRKCB Axis in Diabetic Nephropathy: Integrative Network Pharmacology and Computational Study

**DOI:** 10.1371/journal.pone.0362295

**Published:** 2026-11-05

**Authors:** S. M. Riaduzzaman Niaz, Sumaia Jannat, Sheikh Abdullah Al Ashik, Md. Khairul Islam

**Affiliations:** 1 Department of Biochemistry and Molecular Biology, Mawlana Bhashani Science and Technology University, Tangail, Bangladesh; 2 Department of Biotechnology and Genetic Engineering, Mawlana Bhashani Science and Technology University, Tangail, Bangladesh; University of Sahiwal, PAKISTAN

## Abstract

**Abstract**Diabetic nephropathy (DN), a progressive microvascular complication of diabetes, remains a principal cause of renal failure worldwide, yet its multifaceted molecular complexity continues to challenge effective therapeutic intervention. In this study, an integrative network pharmacology and computational modeling framework was employed to unravel the mechanistic basis of *Terminalia chebula* in mitigating DN pathology. A total of 357 phytoconstituents were retrieved from the IMPPAT 2.0 and PubChem databases and screened through SwissADME and pkCSM, yielding 14 bioactive, drug-like, and non-toxic compounds. Target prediction integrated data from SwissTargetPrediction, BindingDB, and CTD, while DN-related genes were compiled from GeneCards, OMIM, and transcriptomic datasets (GSE154561, GSE154881, GSE217709, and GSE30528). Overlapping targets were analyzed using STRING and Cytoscape to identify key hub proteins, followed by Gene Ontology (GO) and KEGG enrichment analysis. Integration of compound- and disease-associated targets identified 173 overlapping genes. Enrichment analyses revealed significant involvement in the AGE–RAGE signaling pathway, implicating PRKCB as a key regulatory node linking hyperglycemia to oxidative stress, inflammation, and apoptosis. The Random Forest-based QSAR model (R^2^ = 0.91, RMSE = 0.41) denoted Luteolin and Quercetin as potent PRKCB inhibitors (predicted pIC₅₀ ≥ 6). Docking and molecular dynamics simulations confirmed stable PRKCB binding (RMSD ≤ 3.0 Å; ΔG_bind ≈ –122 kcal/mol), supported by DFT analysis indicating high chemical softness and strong charge-transfer potential. This integrative computational analysis suggests that *T. chebula* may exert renoprotective effects through modulation of the PRKCB-driven AGE-RAGE axis, with luteolin and quercetin emerging as promising natural inhibitors. Therefore, these computational findings provide potential molecular insight into the traditional therapeutic efficacy of *T. chebula* and a foundation for further experimental validation in DN.

## 1. Introduction

Diabetes mellitus (DM) is a chronic metabolic disorder that progressively damages multiple organ systems. Among its complications, Diabetic nephropathy (DN) is recognized as a leading cause of end-stage renal disease (ESRD) worldwide [1–3]. DN develops in approximately 30–40% of individuals with type 1 or type 2 diabetes and is a major contributor to increased morbidity and mortality [4]. It is characterized by sustained albuminuria, mesangial matrix expansion, thickening of the glomerular basement membrane, and progressive glomerulosclerosis, ultimately culminating in renal failure [5–7]. The global burden of DN continues to rise in parallel with the increasing prevalence of diabetes, posing significant socioeconomic challenges. Recent epidemiological studies reveal that over 107 million people worldwide were affected by DN in 2021, with mortality and disability-adjusted life years (DALYs) rates increasing significantly over the past three decades despite some declines in age-standardized prevalence rates [8,9]. Projection models indicate that, in the absence of effective interventions, the burden of DN will continue to rise sharply through 2050, particularly affecting low- and middle-income countries with constrained healthcare resources [8]. This alarming trend underscores the urgent need for improved preventive, diagnostic, and therapeutic strategies against DN.

The pathogenesis of DN is multifactorial, involving hemodynamic alterations, chronic hyperglycemia, oxidative stress, inflammation, fibrosis, and apoptosis [10–13]. Despite advances in clinical management through strict glycemic control and renin–angiotensin system (RAS) inhibitors such as angiotensin-converting enzyme inhibitors (ACEIs) and angiotensin receptor blockers (ARBs), current therapeutic options remain insufficient to halt DN progression [14]. Moreover, the long-term use of these agents may cause adverse effects, including hyperkalemia and renal dysfunction, highlighting the urgent need for safer, multi-targeted therapies [14–16].

In recent years, phytochemicals and medicinal plants have drawn considerable attention as alternative or complementary strategies in DN management. Their multitargeted actions, relative safety, and ability to modulate oxidative stress and inflammatory pathways make them attractive candidates for intervention [17–19]. *Terminalia chebula* (Combretaceae), commonly known as Haritaki, is a widely used traditional medicinal plant in Ayurveda, Unani, and other ethnomedical systems. Its fruits are rich in tannins, flavonoids, and phenolic compounds, which exhibit pharmacological activities including antioxidant, anti-inflammatory, antidiabetic, and renoprotective properties [20–22]. Experimental findings indicate that *T. chebula* can improve glycemic control, reduce oxidative damage, and protect renal architecture, suggesting its potential role in DN management [23–26]. However, the precise molecular mechanisms underlying its protective effects remain insufficiently characterized.

To address this gap, computational systems pharmacology has become a powerful tool for elucidating the mechanisms of complex natural products. Unlike traditional “one drug–one target” models, network pharmacology integrates systems-level data to reveal multitarget and multi-pathway mechanisms, which aligns with the therapeutic philosophy of medicinal plants [27]. Molecular docking further validates the binding affinities between bioactive constituents and disease-related targets, offering mechanistic insights at the structural level [28]. Molecular dynamics (MD) simulations complement docking by evaluating the structural stability and conformational dynamics of protein–ligand complexes under physiological conditions. Binding free energy calculations further quantify interaction energetics and strengthen the reliability of docking predictions. In addition, machine learning-based QSAR models predict biological activity from molecular descriptors, providing complementary evidence for compound–target interactions [29–31]. Together, these integrated approaches enable comprehensive exploration of the pharmacological potential of *T. chebula* in DN.

In the present study, we applied a combination of network pharmacology, molecular docking, machine learning-guided quantitative structure–activity relationship (QSAR) modeling, MDS, and post-dynamics MMGBSA analysis to systematically investigate the renoprotective mechanisms of *T. chebula*. This multi-approach strategy not only provides mechanistic insights but also offers a scientific basis for the development of *T. chebula* as a promising natural candidate for DN therapy. The complete workflow of this current study is presented in Fig 1.

**Fig 1:**
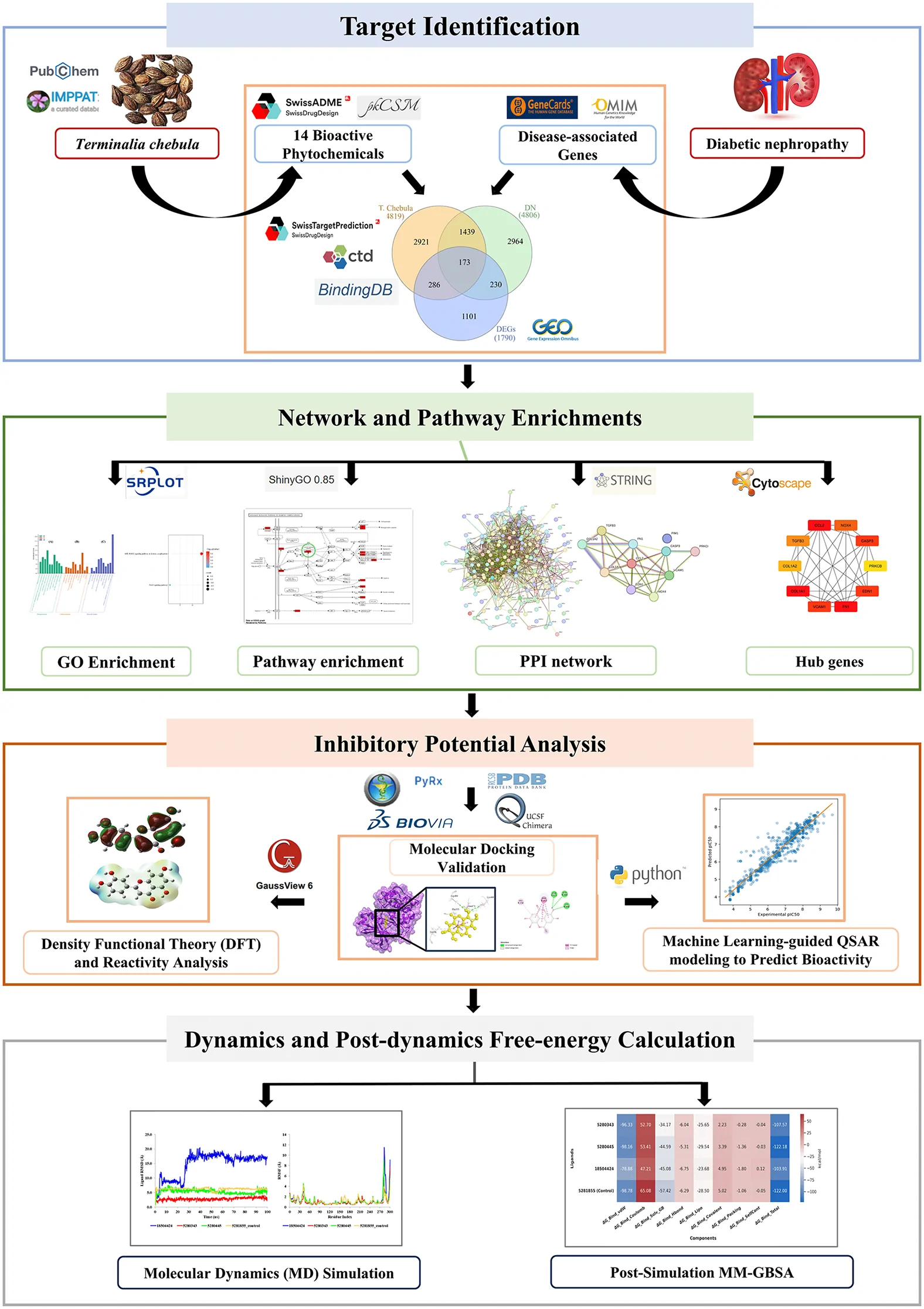
The flow diagram of the multi-approach strategy to unveil mechanistic insights into *T. chebula* against DN.

## 2. Methods and materials

### 2.1. Collection and pharmacokinetic screening of phytoconstituents

The chemical constituents of *T. chebula* were systematically retrieved from the IMPPAT 2.0 database (https://cb.imsc.res.in/imppat/; accessed 16 July 2025) and the PubChem database (https://pubchem.ncbi.nlm.nih.gov/; accessed 16 July 2025). PubChem was selected because it provides a free, publicly accessible, and manually curated repository of chemical information, ensuring accurate structures in canonical SMILES and 3D SDF formats [32]. IMPPAT 2.0 was chosen due to its extensive coverage of traditional Indian medicinal plants, containing data derived from classical texts and thousands of published studies [33]. As *T. chebula* is well represented in this resource, the database provided a reliable basis for assembling a comprehensive compound library. Duplicates were carefully eliminated, and only molecules with complete structural information were retained for further screening.

The SwissADME (http://www.swissadme.ch/; accessed 16 July 2025) and pkCSM (https://biosig.lab.uq.edu.au/pkcsm; accessed 16 July 2025) platforms represent sophisticated computational tools for predicting crucial pharmacological parameters of small molecules. These web-based services offer comprehensive analyses of physicochemical properties, drug-likeness assessments, and ADME (Absorption, Distribution, Metabolism, Excretion) predictions. SwissADME, maintained by the University of Lausanne and the Swiss Institute of Bioinformatics, provides molecular property calculations and drug-likeness evaluations based on established criteria such as Lipinski’s Rule of Five, while pkCSM, developed by researchers including Douglas E. V. Pires and colleagues, employs novel graph-based signatures to generate predictive models for pharmacokinetic properties such as gastrointestinal absorption, cytochrome P_450_ interaction, and P-glycoprotein substrate potential, were also examined [32,34]. Additionally, PAINS filtering was performed using SwissADME, and compounds with >1 PAINS alert were excluded to minimize the risk of assay-interfering and false-positive compounds. Furthermore, safety-related features, including mutagenicity, hepatotoxicity, and skin sensitization, were predicted to eliminate unsuitable candidates. Only compounds fulfilling the majority of drug-likeness and pharmacokinetic criteria were retained for downstream analyses.

### 2.2. Prediction of compound-associated targets

The retrieved phytoconstituents of *T. chebula* were further evaluated for potential molecular targets. Canonical SMILES and structural data of each compound, obtained from PubChem, were utilized for target prediction analyses. Target prediction was performed using three complementary resources to ensure comprehensive coverage. The SwissTargetPrediction platform (http://www.swisstargetprediction.ch/; accessed 17 July 2025) was applied to identify probable protein targets based on both 2D and 3D chemical similarity to known bioactive molecules [35]. To strengthen reliability, the Comparative Toxicogenomics Database (CTD) (https://ctdbase.org/; accessed 17 July 2025) was consulted to extract curated associations between compounds, genes or proteins, and diseases, highlighting potential therapeutic and toxicological relevance [36]. Additionally, the BindingDB database (https://www.bindingdb.org/; accessed 17 July 2025) was used to validate protein-ligand interactions by cross-referencing experimental binding data, applying a similarity score threshold of ≥ 0.9 [36]. Targets consistently predicted across these platforms were retained as candidate compound-associated proteins [22,37].

### 2.3. Prediction of disease-associated targets

Disease-associated targets relevant to DN were retrieved from two well-established genetic and biomedical resources. The GeneCards 5.26 database (https://www.genecards.org/; accessed 17 July 2025) was queried with the term “Diabetic Nephropathy” to obtain a comprehensive list of genes, with higher relevance scores indicating stronger associations [38]. Complementarily, the OMIM database (https://omim.org/; accessed 18 July 2025) was searched to extract curated information on DN-related genes and genetic phenotypes. The intersection between compound-associated targets and DN-related genes was subsequently identified, representing the potential therapeutic targets of *T. Chebula* [39–41].

### 2.4. Identification of differentially expressed genes (DEGs) in DN

To identify genes critically involved in DN, transcriptomic datasets were systematically retrieved from the Gene Expression Omnibus (GEO) database (https://www.ncbi.nlm.nih.gov/geo/; accessed 14 September 2025). Selected datasets included both DN patient samples and healthy controls to ensure disease specificity. Raw expression data were processed and normalized using R-based pipelines, including background correction and quantile normalization, to minimize technical variation. The analysis was performed using the limma package (adjusted p-value < 0.05 and |log2 fold-change| ≥ 1) [42,43]. Only upregulated genes were retained for subsequent analysis, as these genes are more likely to represent activated pathological pathways in DN and potential targets for therapeutic intervention [44].

### 2.5. Integration of compound and disease-associated targets

To explore the potential therapeutic mechanisms of *T. chebula* in DN, phytoconstituent-target prediction was performed using multiple databases, including SwissTargetPrediction, BindingDB, and the Comparative Toxicogenomics Database (CTD). These platforms integrate chemical structure, bioactivity, and literature-based evidence to predict the molecular targets of each compound. The predicted compound-associated targets were then cross-referenced with DN-associated genes, including upregulated DEGs identified from GEO datasets, as well as disease-relevant genes retrieved from GeneCards and OMIM [45–47]. This integration ensured that only targets relevant to both the disease pathology and the phytoconstituents were considered. To facilitate visualization and identification of overlapping targets, interactive Venn diagrams (http://www.interactivenn.net/; accessed 25 September 2025) were employed. Genes present in both datasets were defined as candidate therapeutic targets, representing potential molecular mediators through which *T. chebula* may exert its protective or modulatory effects in DN. This systematic approach reduces false positives and highlights biologically meaningful targets for further network and functional analysis.

### 2.6. Gene Ontology (GO) and pathway enrichment analysis

A comprehensive GO analysis was performed via the DAVID database (https://david.ncifcrf.gov/; accessed 25 September 2025) to elucidate the functional roles of *T. chebula’s* therapeutic targets in DN. The analysis systematically categorized genes according to three primary GO domains: biological processes (BP), revealing disease-related pathways and physiological responses; molecular functions (MF), identifying specific biochemical activities at the molecular level; and cellular components (CC), determining the subcellular localization of target proteins, providing a structured framework to understand how these targets contribute to disease progression at multiple biological levels [48]. Candidate targets identified through the integration of compound-predicted and DN-associated genes were subjected to enrichment analysis in DAVID, and terms with p-value < 0.05 were considered statistically significant to ensure robustness while minimizing false-positive results [49]. For visualization purposes, bubble plots were generated using the SRplot (accessed 26 September 2025) package in R, enabling a clear depiction of the distribution and relative significance of enriched GO terms. In parallel, KEGG pathway enrichment analysis was performed using ShinyGO 0.8 (https://bioinformatics.sdstate.edu/go/; accessed 27 September 2025), which allows systematic mapping of gene sets onto signaling and metabolic pathways and facilitates the identification of biologically relevant networks. Pathways with a threshold of p < 0.05 were retained for further functional interpretation [50]. This integrative analytical approach provides a systematic framework for exploring the potential mechanisms of action of *T. chebula* in DN at multiple biological and molecular levels, establishing a basis for downstream network and mechanistic studies.

### 2.7. Protein-protein interaction (PPI) networks

Protein-protein interaction (PPI) networks are necessary to reveal how proteins work together in complex biological pathways by enabling a deeper understanding of disease mechanisms and identifying potential therapeutic targets [51,52]. The PPI network was generated using the STRING 12.0 database (accessed 30 September 2025), which integrates experimentally validated and computationally predicted protein interactions [53]. A confidence score threshold of 0.4 was applied to ensure balance between reliable interactions and spurious connections. The generated PPI network was imported into Cytoscape v3.10.3 (https://cytoscape.org/; accessed 30 September 2025) for topological analysis: degree, betweenness, and closeness centralities, which were calculated to identify hub proteins, which are nodes that are highly connected and likely critical for network stability and disease progression. These hub proteins may serve as pivotal regulators in DN pathophysiology and potential targets for *T. chebula* phytoconstituents.

### 2.8. Molecular docking studies

Molecular docking plays a vital role in estimating the binding affinity between ligands and target proteins, thereby providing insights into the strength and stability of molecular interactions [54,55]. To investigate the interactions of the selected 14 core compounds of *T. chebula* with the key therapeutic target protein kinase C beta type (PRKCB), the crystal structure of PRKCB was retrieved on 31 October 2025 [56]. The crystal structure of PRKCB (PDB ID: 2I0E) was retrieved from the Protein Data Bank (PDB). The experimentally determined structure was used directly for subsequent docking analysis. Prior to docking, water molecules were removed using BIOVIA Discovery Studio 2020, followed by energy minimization using Swiss-PDB Viewer 4.1.0 with the GROMOS96 43B1 force field to relieve unfavorable steric interactions and optimize the geometry of the retrieved crystal structure. The 3D structures of the core compounds were collected from the PubChem database and prepared in PyRx 0.8 (accessed 1 November 2025) for conversion into PDBQT format. Docking studies were initially performed using AutoDock VINA via PyRx, where a grid box was precisely centered on the active site of PRKCB with dimensions X = 20.6542 Å, Y = 13.7961 Å, and Z = 18.9644 Å to ensure accurate coverage of the binding pocket. The centroid coordinates were selected to coincide with the center of the reported catalytic binding pocket, while the grid dimensions were optimized to fully encompass the active-site residues and provide sufficient space for ligand flexibility during docking. Binding affinities were expressed as docking scores in kcal/mol, with more negative values representing stronger binding interactions [57]. Visualization of complexes and identification of key hydrogen bonding and hydrophobic interactions were carried out using Chimera and BIOVIA Discovery Studio Visualizer [58,59]. To ensure reliability and improve accuracy, the top-ranked conformations from AutoDock VINA were further validated using GLIDE docking (Schrödinger 2024−2, Extra Precision mode, accessed 11 November 2025) [60]. GLIDE docking was performed with default XP parameters to validate docking poses and assess the stability of ligand binding within the PRKCB active site [61,62]. Moreover, the docking method was validated using DockRMSD [63]. The docking scores obtained from GLIDE confirmed the binding trends observed in AutoDock VINA, thereby reinforcing the consistency and robustness of the computational predictions.

### 2.9. QSAR model performance and identification of potential inhibitors

QSAR modeling provides insights into the relationship between molecular structure and biological activity, enabling the prediction of compound properties and activity based on molecular descriptors [64]. To complement the molecular docking approach and gain deeper insight into the inhibitory potential of *T. chebula* phytoconstituents against PRKCB, a QSAR-based machine learning framework was developed. Bioactivity data specific to PRKCB were collected from the ChEMBL 36 database (Target ID: ChEMBL3045; accessed 13 November 2025), which contains 4535 compounds from different bioassays. The focus on compounds was limited to 878 inhibitors with reported IC_50_ values. These values were standardized and transformed into their negative logarithmic form (pIC_50_) to allow direct comparison of inhibitory potency across compounds. Based on widely accepted thresholds, molecules with pIC_50_ values greater than or equal to 6 were considered active, while those with pIC_50_ values of 5 or lower were categorized as inactive compounds. The intermediate range was excluded to avoid bias and misclassification [65].

The chemical structures of the collected molecules, expressed as canonical SMILES, were analyzed using the PaDEL-Descriptor toolkit, which generated an extensive set of molecular descriptors and structural fingerprints [66]. These descriptors captured both physicochemical characteristics and substructural features, including Lipinski’s Rule of Five parameters. To reduce noise and redundancy, descriptors exhibiting high correlation were removed, ensuring a refined and non-collinear dataset suitable for model building.

The curated dataset was divided into a training set (80%) for model construction and a test set (20%) for external validation. A Random Forest regression algorithm was selected due to its ability to manage non-linear relationships and large descriptor spaces while minimizing overfitting [50,67]. The number of trees was fixed at 100, with default values applied for the remaining hyperparameters. Model performance was assessed by calculating the coefficient of determination (R^2^), which reflects the proportion of variance explained, and the root mean square error (RMSE), which measures the accuracy of predictions. According to established QSAR validation criteria, models were regarded as acceptable when R^2^ exceeded 0.6 for the training set and 0.5 for the test set. Scatter plots comparing predicted versus experimental pIC_50_ values were also generated to provide a visual indication of model reliability. Finally, the optimized model was integrated into an interactive Streamlit web application to facilitate user-friendly predictions. The application is available at (https://github.com/Niaz4020/Bioactivity-Prediction-App-For-PRKCB-.git) to predict the activity of new compounds and reproduce the results.

### 2.10. Density functional theory (DFT) calculations

Density Functional Theory (DFT) calculations provide insights into the electronic structure, molecular stability, reactivity, and quantum-chemical properties of molecular systems [68]. The quantum chemical analysis was performed using the Gaussian 09 software package based on DFT [69]. GaussView 6.0 (accessed 20 November 2025) was employed for visualization purposes, aiding in the creation of input files and the subsequent generation of graphical representations of the compounds [70]. Electronic structure calculations were implemented through DFT methodology, employing the B3LYP method with the 6-31G(d,p) basis set. This computational approach integrates Becke’s three-parameter exchange functional with the Lee-Yang-Parr correlation scheme, enabling precise electronic structure characterization [71–73]. The top-performing ligands, identified through docking studies, were further analyzed using DFT. This analysis focused on frontier molecular orbitals (HOMO-LUMO), energy gaps, and molecular electrostatic potential (MEP) maps. The MEP maps, generated from optimized geometries and visualized with GaussView 6.0, were used to analyze charge distribution [74]. The frontier orbitals play a critical role in determining the electronic properties and reactivity of molecules. The energies of the HOMO and LUMO orbitals were employed to estimate key reactivity descriptors, namely chemical hardness (η) and chemical softness (S). In a chemical context, hardness refers to the resistance of an atom or molecule to the transfer of electrons, either by donation or acceptance from other atoms or metallic surfaces, while softness denotes the ease with which such electron transfer can occur [75]. The chemical hardness (η) and softness (S) of the compound were determined using the equations derived from the Parr–Pearson interpretation of DFT in conjunction with Koopmans’ theorem [76,77].


$$\begin{array}{c} \eta =\frac{\varepsilon_{HOMO-}\varepsilon_{LUMO}\;}{\text{2}} \end{array}$$ (1)

$$\begin{array}{c} s=\frac{\text{1}}{\eta } \end{array}$$ (2)

Greater reactivity indicates lower hardness (η) and a higher softness (S) value, while a higher hardness (η) and lower softness (S) values indicate reduced reactivity [78]. S measures an atom’s ability to accept electrons and is inversely proportional to η.

### 2.11. Molecular dynamics (MD) simulations

To validate the molecular docking predictions and explore the dynamic stability of the ligand-protein complexes [79,80], MD simulations were conducted for three major phytochemicals, Luteolin (CID: 5280445), Quercetin (CID: 5280343), and 3,4,8,9,10-Pentahydroxydibenzo(b,d)pyran-6-one (CID: 18504424), alongside the standard inhibitor Ellagic Acid (CID: 5281855). The molecular dynamics (MD) simulations were conducted utilizing the Desmond module within Maestro 2025.1 (Schrödinger Suite) on a Linux-based system (Ubuntu 22.04). Each protein-ligand complex was embedded in an orthorhombic box and solvated with TIP3P water molecules, maintaining a minimum solvent buffer of a:10 Å, b: 10 Å, c: 10 Å to mitigate periodic boundary effects. The systems were neutralized and brought to physiological ionic strength (0.15 M) through the addition of Na⁺ and Cl^-^ counterions. A 12 Å spherical cutoff was implemented for long-range electrostatic interactions to optimize computational efficiency. System parameterization was performed using the OPLS_2005 force field. Following energy minimization, the systems underwent systematic equilibration protocols. Production simulations were executed under isothermal-isobaric (NPT) ensemble conditions, with temperature regulation at 310 K via the Nose-Hoover thermostat and pressure maintenance at 1.01325 bar through isotropic coupling. Each simulation was extended to achieve a 100 ns trajectory. System stability was assessed through root mean square deviation (RMSD), while flexibility was monitored using root mean square fluctuation (RMSF). Additional parameters, including radius of gyration (Rg), solvent-accessible surface area (SASA), hydrogen bond occupancy, polar surface area (PSA), and molecular surface area (MolSA), were quantified to characterize conformational stability and compactness of the complexes.

### 2.12. Binding free energy calculations

Binding free energy (ΔG_bind) was calculated using the molecular mechanics–generalized Born surface area (MM/GBSA) methodology [81]. Calculations were carried out with the thermal_mmgbsa.py utility of the Schrödinger suite, applying the OPLS_2005 force field. Representative frames extracted from the 100 ns MD trajectory were analyzed to obtain ensemble-averaged values describing the thermodynamic stability of the ligand–protein complex. The binding free energy was evaluated using the following thermodynamic relationship:


$$\begin{array}{c} △G_{bind}=G_{complex}-(G_{protein}+G_{ligand}) \end{array}$$ (3)

which can be decomposed as:


$$\begin{array}{c} △G_{bind}=△E_{MM}\;+\;△G_{GP}\;+\;△G_{SA} \end{array}$$ (4)

where ΔE_MM_ represents bonded, electrostatic, and van der Waals interactions, ΔG_GB_ accounts for polar solvation energies derived from the Generalized Born model, and ΔG_SA_ corresponds to nonpolar solvation contributions from solvent-accessible surface area. Average ΔG_bind_ values across the 100 ns trajectories were considered for final interpretation, thereby providing quantitative insights into the stability and energetics of ligand-PRKCB complexes.

## 3. Results

### 3.1. Pharmacokinetic and safety profiling of bioactive phytoconstituents

A total of 357 phytoconstituents of *T. chebula* were initially collected from the IMPPAT 2.0 and PubChem databases, reflecting the plant’s chemical richness. After duplicate removal and structural verification, all compounds were subjected to *in silico* pharmacokinetic and safety screening to identify molecules with favorable drug-like properties. Screening parameters included drug-likeness rules (Lipinski, Veber, Ghose), oral bioavailability, gastrointestinal absorption, metabolic stability (CYP interactions), P-glycoprotein substrate potential, and toxicity predictions (AMES, hepatotoxicity, skin sensitization, and hERG inhibition). Compounds failing multiple criteria were excluded to ensure only safe and pharmacologically relevant molecules were retained (S1 Table).

Following this multistage screening, 14 compounds were selected as bioactive candidates (Table 1). All 14 compounds exhibited high gastrointestinal absorption. None were P-glycoprotein substrates, and only a few showed minor skin sensitization, indicating a favorable safety profile. Metabolic analysis suggested selective interactions with cytochrome P450 isoforms, indicating potential metabolic modulation without broad toxicity risk. The selected compounds include flavonoids (Quercetin, Luteolin), terpenoids (Squalene), and fatty acid derivatives, displaying a wide range of molecular weights (172–410 g/mol), lipophilicity (Log P 0.66–3.93), and polar surface areas (17–131 Å²). This structural diversity suggests the ability to interact with multiple molecular targets, aligning with the multi-component, multi-target therapeutic potential of *T. chebula*.

**Table 1:** *In silico* pharmacokinetic and safety screening of 14 *T. chebula* phytoconstituents, highlighting drug-likeness, absorption, metabolic profile, and toxicity for selection of bioactive candidates.

PubChem CID	Compounds	MW	GIA	HT	PA
2969	Capric Acid	172.26	High	No	Antibacterial and anti-inflammatory activity [82,83]
8163	2-Undecanone	170.29	High	No	Anti-inflammatory; antimicrobial/antifungal activity [84,85]
26479	Carbamic acid, methyl-, 3-(dimethylamino)-p-tolyl ester	208.26	High	No	Not specifically reported
95495	6,10-Dimethylundecan-2-one	198.34	High	No	Not specifically reported
638072	Squalene	410.72	High	No	Antioxidant and anti-inflammatory; also anti-atherosclerotic and antineoplastic effects reported [86–88]
5280343	Quercetin	302.24	High	No	Antioxidant, anti-inflammatory, anti-fibrotic and renoprotective /anti-DN activity [89–91]
5280445	Luteolin	270.24	High	No	Antioxidant, anti-inflammatory, antidiabetic, anticancer, antimicrobial, antiviral, neuroprotective and other activities [92–94]
5317247	Ethyl geranate	196.29	High	No	Antimicrobial and anti-inflammatory activities [95]
5469252	Phenol, (Z)-	226.27	High	No	Antioxidant and antimicrobial activities [96]
9904352	2-(3-Ethyl-4-oxo-5-piperidino-2-thiazolidinylidene) acetic acid ethyl ester	298.4	High	No	Not specifically reported
11107141	2-[(Carboxymethyl)amino]-4-chlorobenzoic acid	229.62	High	No	Not specifically reported
11252659	12-Deuteriododecanoic acid	201.32	High	No	Not specifically reported
18504424	3,4,8,9,10-Pentahydroxydibenzo(b,d)pyran-6-one	276.2	High	No	Antioxidant and anti-inflammatory activity [22,37]
52914864	(3E,9E)-undeca-3,9-dienoic acid	182.26	High	No	Not specifically reported

*Note: MW = Molecular Weight, GIA = Gastrointestinal Absorption, HT = Hepatotoxicity, PA = Pharmacological Activity.*

### 3.2. Prediction of compound-associated and disease-relevant targets

The 14 bioactive compounds of *T. chebula* were screened to identify their potential molecular targets using SwissTargetPrediction, BindingDB, and the Comparative Toxicogenomics Database (CTD). This analysis yielded 4,819 unique targets (S2 Table), with flavonoids such as quercetin and luteolin contributing the largest share, consistent with their known multi-target pharmacological activity.

In parallel, disease-associated targets relevant to DN were retrieved from the GeneCards and OMIM databases. After filtering for disease relevance and removing duplicates, a total of 4,806 DN-related genes were obtained. To further refine candidate targets implicated in DN pathophysiology, four transcriptomic datasets (GSE154561, GSE154881, GSE217709, and GSE30528) were systematically analyzed. Differential expression analysis identified 4,885 DEGs between DN and control samples, comprising 1,790 upregulated (in red) and 3,095 downregulated (in blue) genes (**Fig 2A-D**). Given their higher probability of driving pathogenic processes, only upregulated DEGs were retained for subsequent analyses. Integration of these datasets was performed using the InteractiVenn tool, intersecting *T. chebula* compound-associated targets, DN-related genes, and DEGs. This approach yielded 173 overlapping genes, representing the most likely molecular mediators through which *T. chebula* exerts therapeutic effects in DN (Fig 2E).

**Fig 2:**
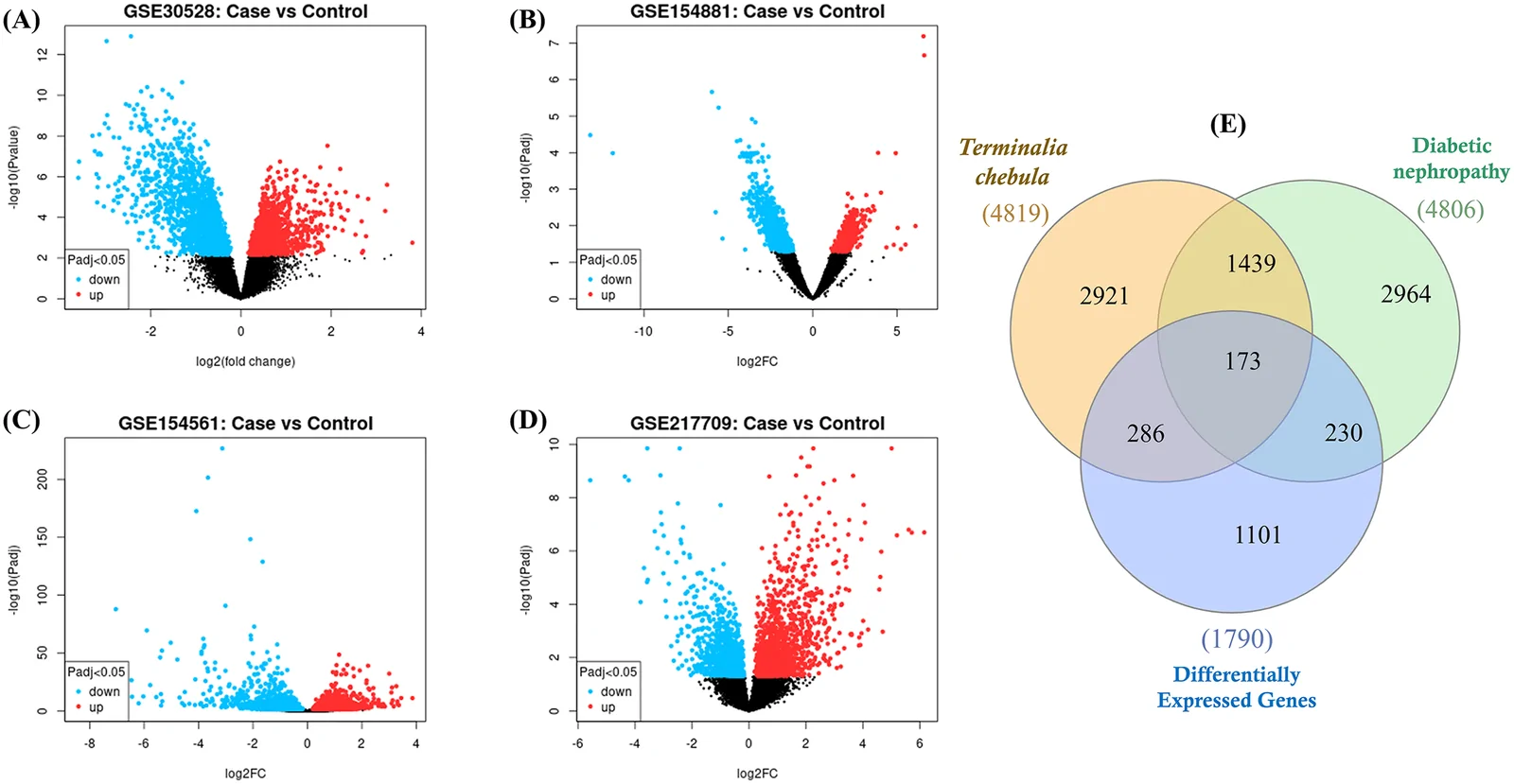
Differential expression analysis of four GEO datasets. **(A)** GSE154561, **(B)** GSE154881, **(C)** GSE217709, and **(D)** GSE30528 identified significantly upregulated (red) and downregulated (blue) genes in DN. Integrative analysis using InteractiVenn **(E)** revealed 173 overlapping targets between *T. chebula* bioactive compound–associated targets, DN-related genes, and upregulated DEGs, representing key candidate mediators of its therapeutic action.

### 3.3. GO and enrichment analysis

The biological significance of the overlapping targets was explored through GO enrichment analysis (S3 Table), with annotations organized into three principal domains: BP, CC, and MF (Fig 3A-C). In the BP category, the enriched targets were predominantly associated with pathways relevant to DN, including inflammatory response, positive regulation of PI3K/AKT signaling, positive regulation of canonical NF-κB signaling, cellular response to tumor necrosis factor, and regulation of apoptosis. Processes such as negative regulation of angiogenesis and response to xenobiotic stimulus were also significantly enriched, indicating a strong link to oxidative stress and inflammation in disease progression. The CC analysis revealed that most of the candidate targets were localized in the extracellular space, extracellular exosome, collagen-containing extracellular matrix, and external side of the plasma membrane. These findings suggest that the therapeutic actions of *T. chebula* compounds may be mediated through extracellular signaling and matrix remodeling. In the MF category, enriched terms included protein binding, identical protein binding, cytokine activity, integrin binding, extracellular matrix structural constituent, and signaling receptor binding. Additionally, enzyme-related activities such as serine-type endopeptidase activity and cysteine-type endopeptidase inhibitor activity involved in apoptosis were significantly represented, underscoring the regulatory role of these targets in inflammation and programmed cell death.

**Fig 3:**
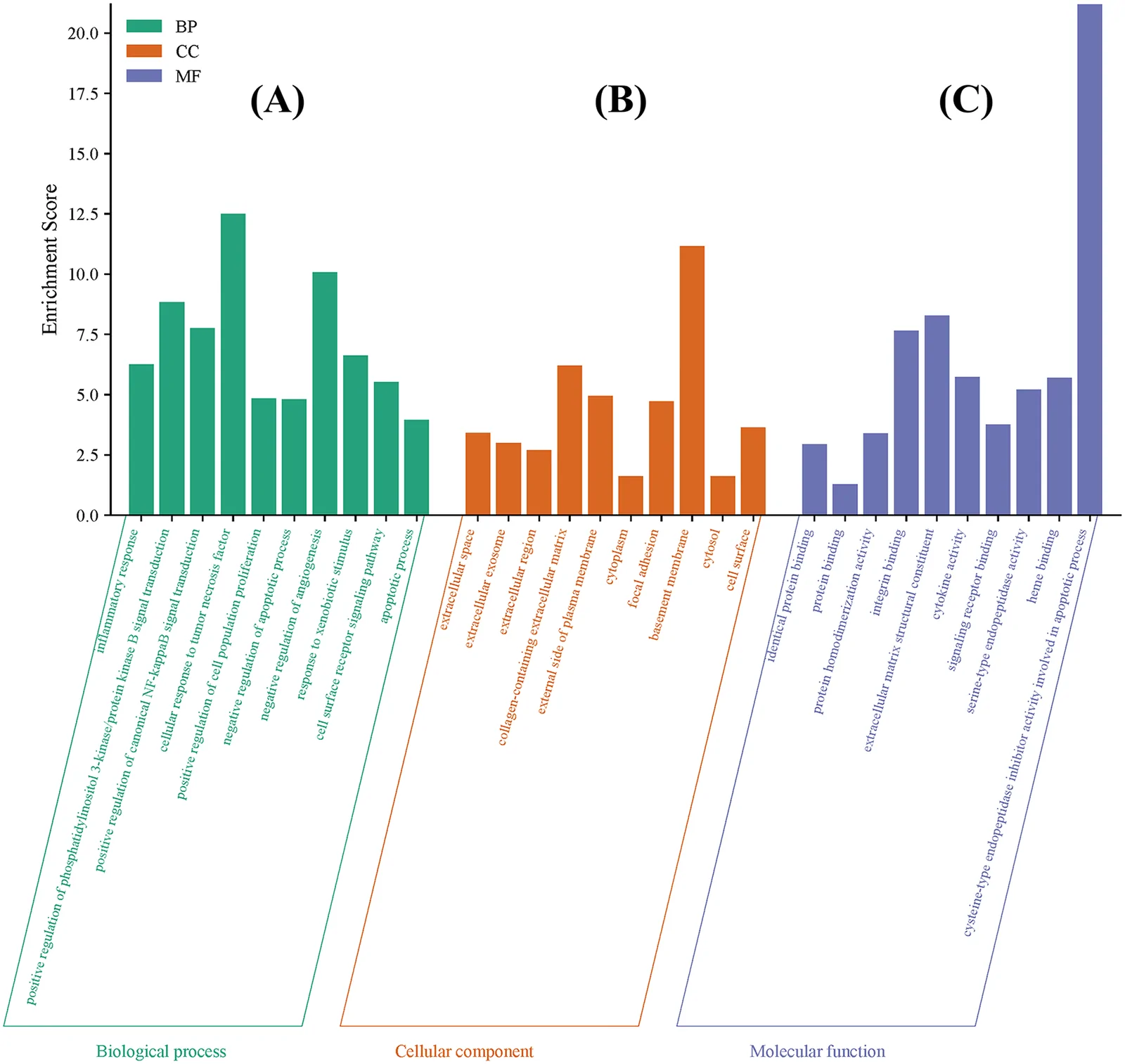
Gene Ontology (GO) enrichment analysis of overlapping targets categorized into (A) Biological Process (BP), (B) Cellular Component (CC), and (C) Molecular Function (MF).

### 3.4. KEGG pathway enrichment and the AGE-RAGE axis

To explore the molecular mechanisms through which *T. chebula* may exert protective effects in DN, we performed KEGG pathway enrichment analysis on the 173 overlapping targets derived from compound-target predictions and DN-associated genes. From the KEGG DN pathway database (https://www.genome.jp/dbget-bin/www_bget?H01456), four canonical DN-related pathways (AGE-RAGE signaling pathway in diabetic complications, the FoxO signaling pathway, the renin–angiotensin system, and the longevity regulating pathway) were considered. However, enrichment analysis provided insights that only two were significantly represented in our dataset: the AGE-RAGE signaling pathway in diabetic complications and the FoxO signaling pathway (Fig 4).

**Fig 4:**
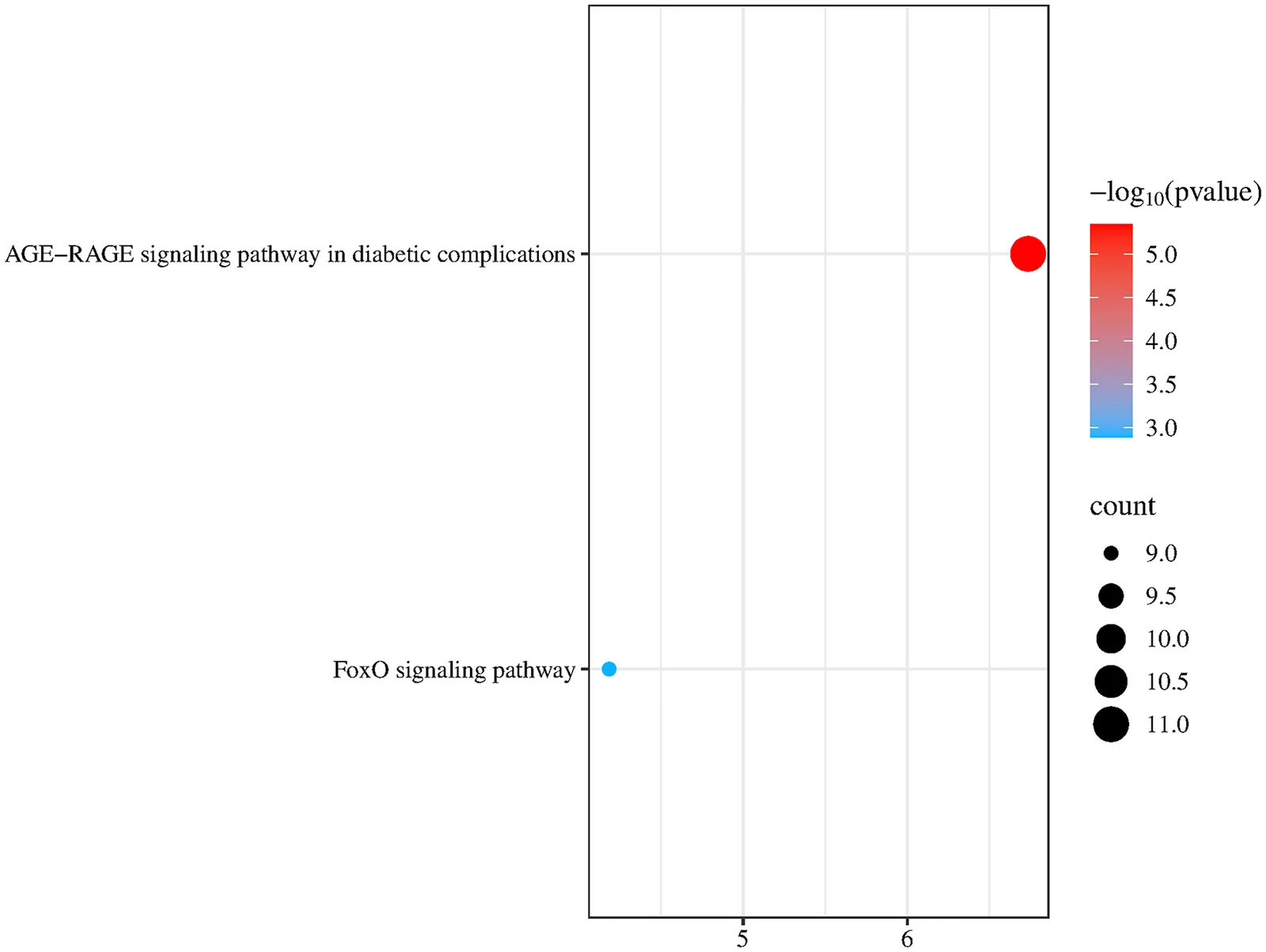
Enriched KEGG pathways of *T. chebula* targets overlapping with DN genes, indicating the AGE-RAGE signaling pathway in diabetic complications.

Among these, the AGE-RAGE signaling pathway showed the strongest enrichment, supported by a high gene count and the most significant *p*-value (−log10 *p* > 5). This pathway has been widely implicated in diabetic complications, where hyperglycemia-driven accumulation of advanced glycation end products (AGEs) activates the receptor for AGE (RAGE), initiating cascades of oxidative stress, inflammation, and fibrotic remodeling [97,98]. The enrichment of this pathway strongly suggests that *T. chebula* phytoconstituents may exert protective effects in DN by attenuating AGE–RAGE–mediated cellular injury. The FoxO signaling pathway was also enriched, though with lower statistical significance (−log10 p ≈ 3). FoxO transcription factors are key regulators of oxidative stress responses, apoptosis, and glucose metabolism [99,100], suggesting that *T. chebula* may additionally enhance stress resilience and metabolic balance in DN through this axis.

### 3.5. PPI network and Hub genes

A total of 173 candidate genes were identified at the intersection of *T. chebula* compound targets and differentially expressed genes in DN. Implementing a medium confidence score threshold (0.4), the analysis yielded a comprehensive interaction network comprising 170 protein nodes interconnected by 1059 functional associations (Fig 5A). This dense interaction network indicates that the candidate genes are functionally linked and may participate in shared biological processes relevant to DN progression.

**Fig 5:**
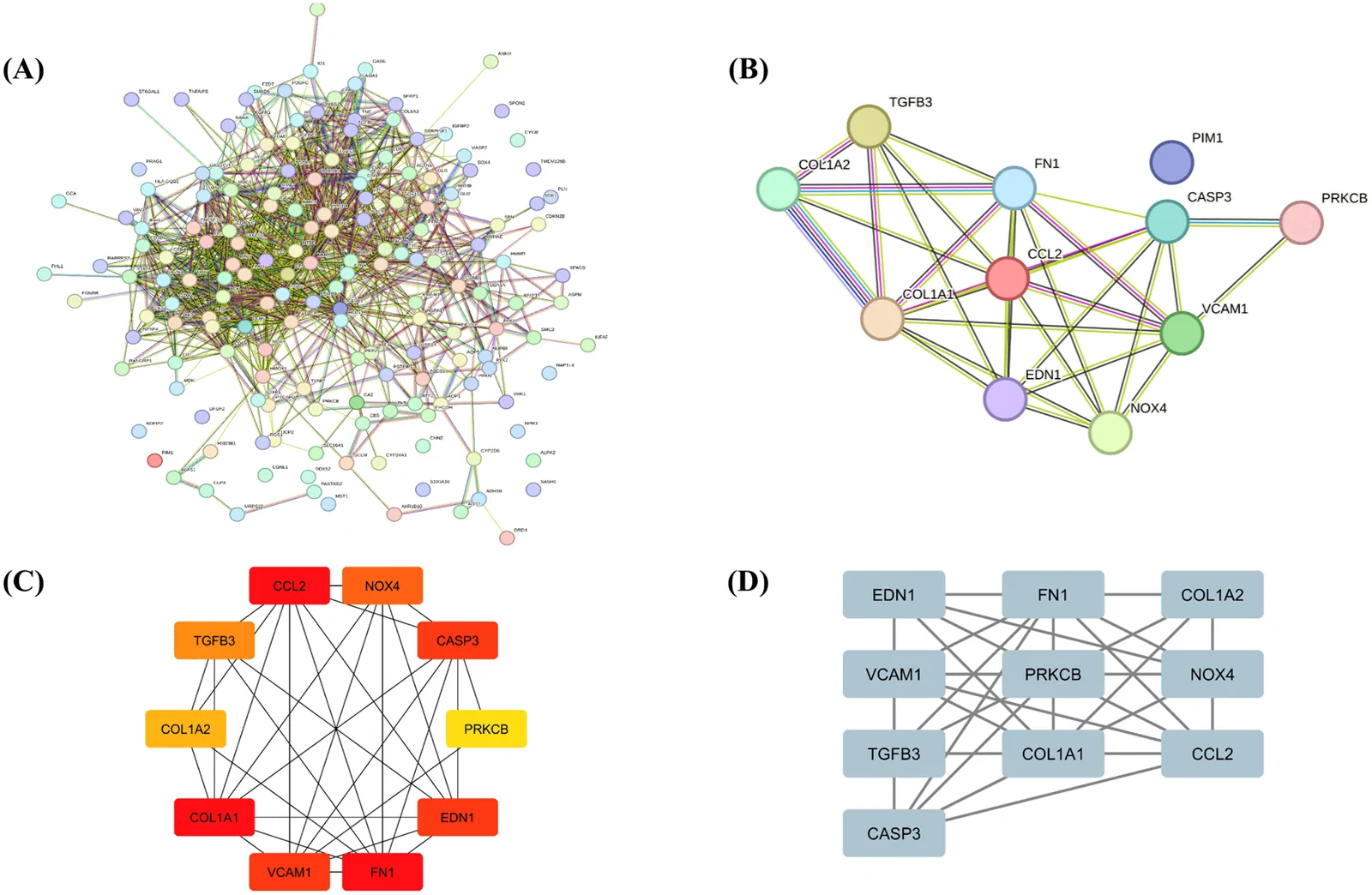
Protein-protein interaction (PPI) network of the 173 overlapping targets associated with *T. chebula* and DN. **(A)** Global PPI network constructed in STRING **(B)**. Focused PPI subnetwork of 11 genes enriched in the AGE-RAGE signaling pathway, highlighting their potential involvement in DN pathogenesis. **(C)** Hub gene identification based on degree, betweenness, and closeness centrality. **(D)** Cluster analysis of the AGE–RAGE pathway genes in Cytoscape.

As the AGE–RAGE signaling pathway in diabetic complications emerged as the most significantly enriched pathway in the earlier KEGG analysis, it was selected for focused investigation. To validate and refine this finding, the KEGG pathway enrichment option in STRING was examined using the 170 networked genes. This revealed that 11 genes were directly associated with the AGE–RAGE pathway. These genes were then extracted to construct a pathway-specific PPI subnetwork to better understand their internal connectivity and potential cooperative roles (Fig 5B). The resulting subnetwork, consisting of 11 nodes and 36 edges, illustrated strong molecular interactions, suggesting that these targets act together in regulating key pathological mechanisms such as oxidative stress, inflammation, extracellular matrix remodeling, and apoptosis processes that are central to DN pathogenesis.

To identify critical regulatory nodes within this subnetwork, topological analysis was performed using Cytoscape based on degree, betweenness, and closeness centrality parameters. The integrated results highlighted PRKCB, CCL2, COL1A1, VCAM1, FN1, CASP3, NOX4, TGFB3, COL1A2, and EDN1 (Table 2) as highly connected and topologically significant hub genes (Fig 5C). These hubs likely play essential roles in coordinating the AGE–RAGE–driven signaling network and may serve as key molecular mediators of *T. chebula*’s renoprotective actions. Furthermore, clustering analysis of the subnetwork revealed functionally coherent modules, indicating that these genes operate in tightly associated sub-networks to regulate overlapping biological processes (Fig 5D). Mapping of the enriched targets to this pathway identified 11 genes (red) distributed across key pathogenic processes, including oxidative stress, inflammation, extracellular matrix remodeling, and apoptosis (Fig 6) [101]. Among these genes, PRKCB (Protein Kinase C beta), a prominent isoform of the PKC family, emerged as a pivotal hub integrating AGE–RAGE activation with multiple downstream cascades such as NF-κB, MAPK/ERK, and PI3K–Akt signaling [19,82–84]. Previous experimental studies have also demonstrated that abnormal PRKCB activation accelerates glomerular damage and renal fibrosis [102–104]. Therefore, inhibition of PRKCB represents a promising therapeutic strategy for slowing the progression of DN.

**Table 2:** The top 10 protein-protein interactions associated with the AGE–RAGE pathway.

Protein name	Degree centrality	Betweenness centrality	Closeness centrality
CCL2	8	5.1	8.5
FN1	8	5.1	8.5
COL1A1	8	5.1	8.5
VCAM1	7	7	8
CASP3	7	7	8
EDN1	7	2	8
NOX4	6	0	7.5
TGFB3	5	0.5	6.8
COL1A2	4	0	6.3
PRKCB	2	0	5.1

**Fig 6:**
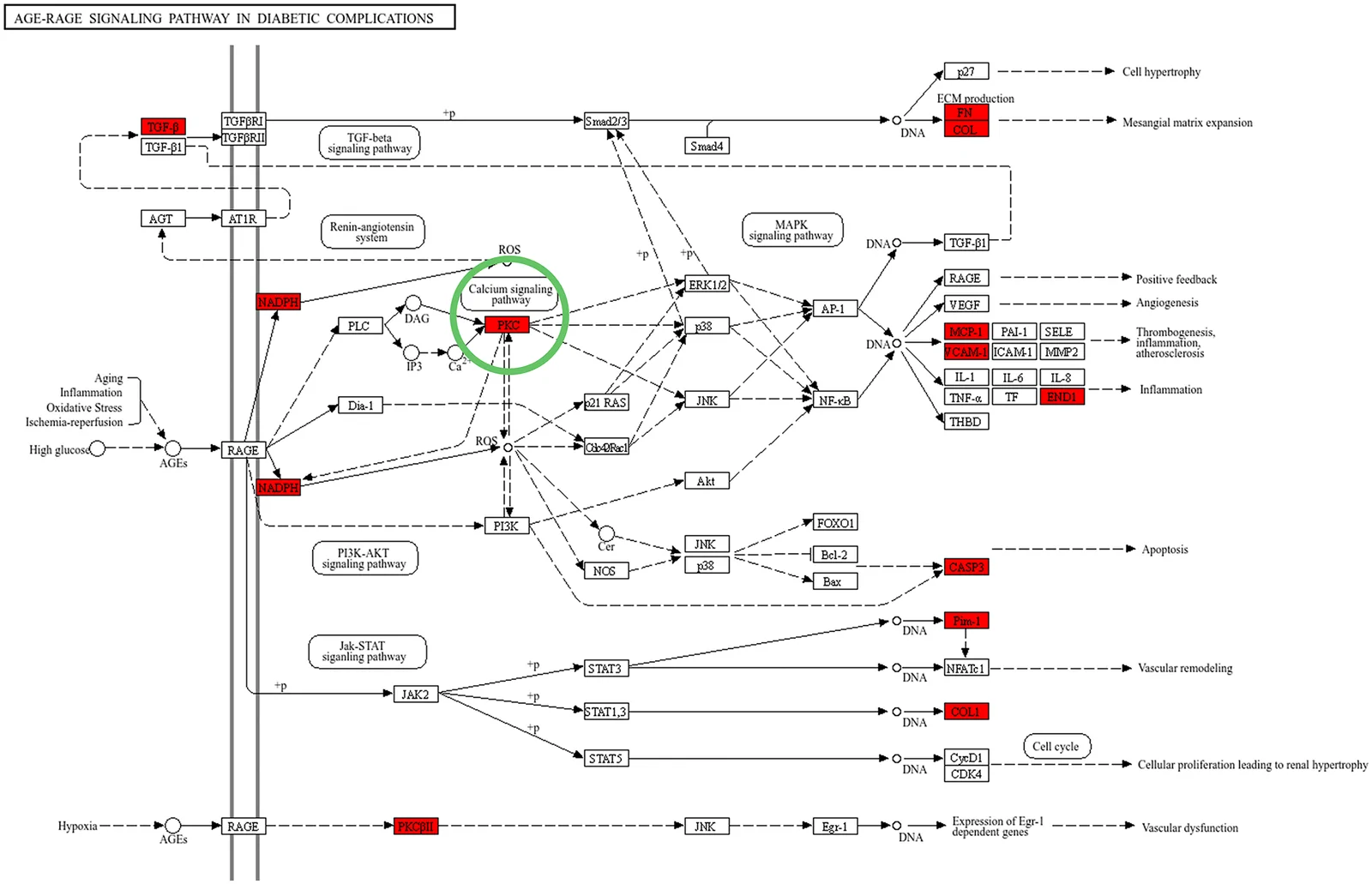
AGE-RAGE signaling pathway highlighting the potential 11 molecular targets modulated by *T. chebula* (indicated in red). Reprinted from the KEGG Pathway Database (map04933) under a CC BY 4.0 license, with permission from Kanehisa Laboratories, original copyright © [2026].

### 3.6. Molecular docking of PRKCB and *Terminalia chebula* phytochemicals

Molecular docking simulations were performed using AutoDock VINA to evaluate the binding potential of selected *T. chebula-*derived phytoconstituents against protein kinase C beta (PRKCB), a critical therapeutic target in DN [105,106]. The structure of the compounds is presented in Fig 7. All compounds were docked into the same binding pocket of PRKCB (PDB ID: 2I0E) under optimized and consistent docking parameters (Fig 8). The docking scores of the candidate ligands, along with the control compound Ellagic acid, ranged between −7.4 and −8.3 kcal/mol, with an RMSD of 0 considered for scoring to ensure reliability (S4 Table). The hydrogen-bonding and hydrophobic interaction profiles presented in Table 2 were derived from the highest-ranked AutoDock Vina docking conformations, while Glide XP docking was subsequently employed as an independent validation approach to confirm the consistency of binding affinities and ligand-binding poses. Among the 14 compounds, three displayed the most favorable interactions (Table 3), with luteolin emerging as the strongest binder. Luteolin exhibits docking energies of −8.3 kcal/mol (AutoDock VINA) and −9.25 kcal/mol (GLIDE). It established multiple hydrogen bonds with LYS371, GLU390, and ASP484, and additional hydrophobic contacts with LEU348, VAL356, ALA369, MET420, and ALA483, providing strong stabilization within the catalytic site (Fig 8A). Quercetin also exhibited robust binding, with energies of −7.8 kcal/mol (AutoDock VINA) and −9.93 kcal/mol (GLIDE). Its stability was reinforced through hydrogen bonds with LYS371, GLU390, ASP470, ASP484, and PHE485, alongside hydrophobic interactions with VAL356, ALA369, MET420, MET473, and ALA483 (Fig 8B). Similarly, 3,4,8,9,10-pentahydroxydibenzo(b,d)pyran-6-one yielded docking energies of −7.4 kcal/mol (AutoDock VINA) and −9.41 kcal/mol (GLIDE), forming hydrogen bonds with PHE353, LYS371, and GLU390, complemented by hydrophobic interactions with VAL356, MET420, and ALA483 (Fig 8C). The control molecule, Ellagic acid, exhibited binding affinities of −7.8 kcal/mol (AutoDock VINA) and −8.53 kcal/mol (GLIDE), interacting through hydrogen bonds with LYS468, ASN471, and ASP484, and hydrophobic interactions with PHE353 and VAL356 (Fig 8D). The co-crystallized ligand 3-{1-[3-(Dimethylamino)propyl]-2-Methyl-1h-Indol-3-Yl}-4-(2-Methyl-1h-Indol-3-Yl)-1h-Pyrrole-2,5-Dione exhibited docking scores of −8.9 kcal/mol (AutoDock Vina) and −9.2 kcal/mol (Glide), stabilized by hydrogen bonding with Asp470 and Phe485, and hydrophobic interactions with Leu348, Phe353, Val356, Ala369, Lys371, Leu394, Met420, and Ala483 (Fig 8E). Docking pose validation is presented in Fig 8F, where the calculated docking RMSD is 0.424 (< 2 Å), confirming a validated docking procedure. Although co-crystallized ligand binding affinity is comparable, both AMES and Hepatotoxicity exist in the case of the co-crystallized ligand. Importantly, most of the interacting residues identified in this study correspond to the previously reported active binding pocket of PRKCB (LYS371, GLU390, ASP466, ASN471, and ASP484), further supporting the reliability of these docking results [104]. Moreover, these results suggest that luteolin and quercetin exhibit stronger binding within the catalytic pocket of PRKCB compared to the reference inhibitor, underscoring their potential role as natural modulators of PRKCB activity in the context of DN.

**Table 3:** Molecular Docking Binding Affinities and Key Interactions of *T. chebula* phytoconstituents with PRKCB. Interaction profiles were obtained from AutoDock Vina, while Glide XP scores were included for validation.

Compound	PubChem CID	Binding affinity (kcal/mol)		Hydrogen bond	(Distance H-A (Å)	Hydrophobic Interaction	Distance (Å)
**AutoDock VINA**	**GLIDE**
Luteolin	5280445	−8.3	−9.25	LYS371 GLU390 ASP484	2.58 2.39 2.26	LEU348 VAL356 ALA369 MET420 ALA483	5.43 4.57 4.52 5.49 4.62
Quercetin	5280343	−7.8	−9.93	LYS371 GLU390 ASP470 ASP484 PHE485	2.78 2.08 2.14 2.73 2.35	VAL356 ALA369 MET420 MET473 ALA483	4.83 5.37 4.77 5.45 4.62
3,4,8,9,10-Pentahydroxydibenzo(b,d)pyran-6-one	18504424	−7.4	−9.41	PHE353 LYS371 GLU390	2.98 2.64 1.99	VAL356 MET420 ALA483	5.17 5.43 5.00
Ellagic Acid (Control)	5281855	−7.8	−8.53	LYS468 ASN471 ASP484	2.95 2.29 2.70	PHE353 VAL356	3.64 5.10
Co-crystalized ligand	11963533	−8.9	−9.2	ASP470 PHE485	2.69 3.34	LEU348 PHE353 VAL356 ALA369 LYS371 LEU394 MET420 ALA483	4.69 3.90 3.89 4.43 4.49 5.47 4.46 5.10

**Fig 7:**
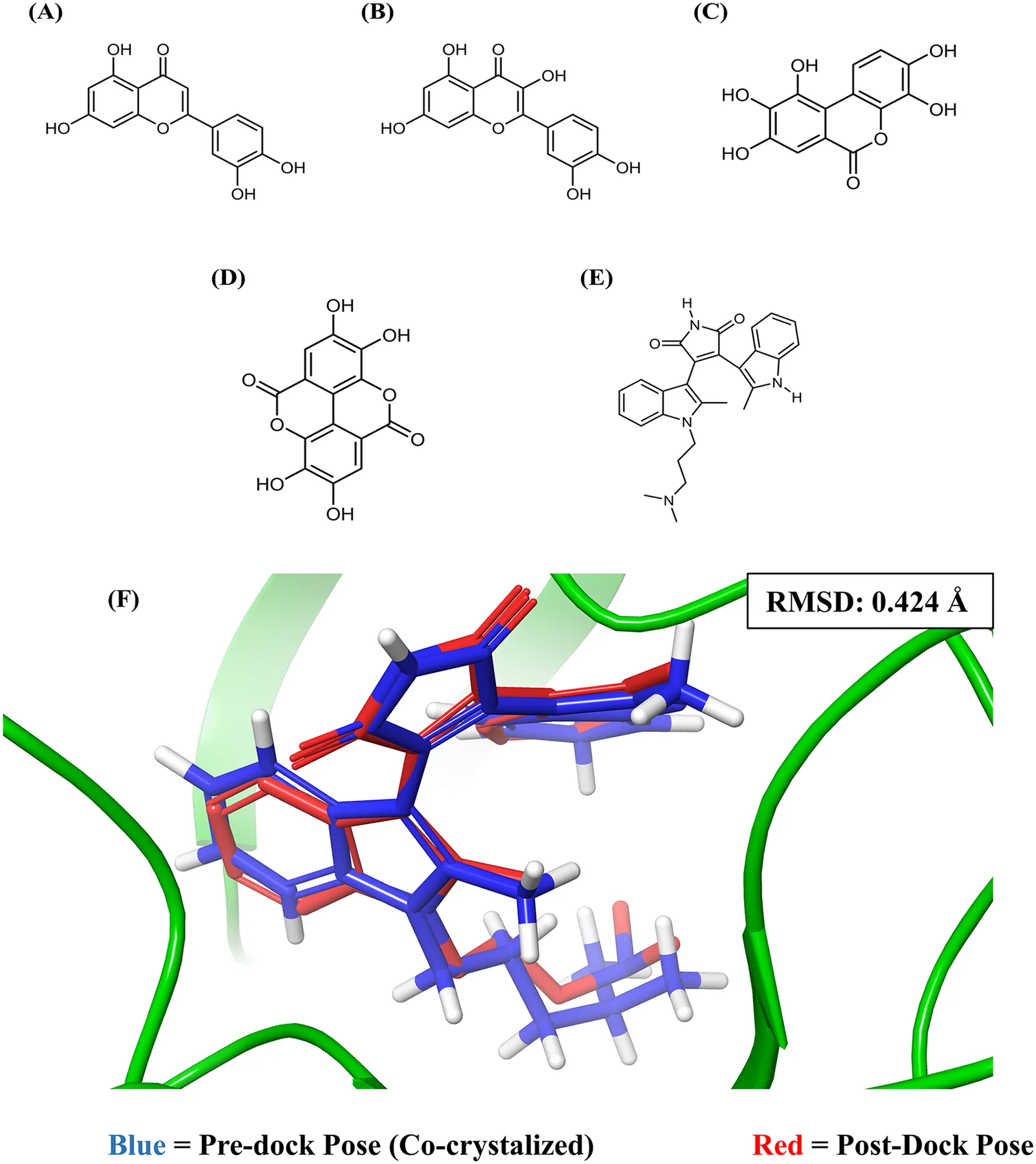
Chemical structures of (A) Luteolin, (B) Quercetin, (C) 3,4,8,9,10-Pentahydroxydibenzo(b,d)pyran-6-one, (D) Ellagic Acid (Control) by using ChemDraw and (E) Co-crystalized ligand 3-{1-[3-(Dimethylamino)propyl]-2-Methyl-1h-Indol-3-Yl}-4-(2-Methyl-1h-Indol-3-Yl)-1h-Pyrrole-2,5-Dione, (F) Docking pose validation.

**Fig 8:**
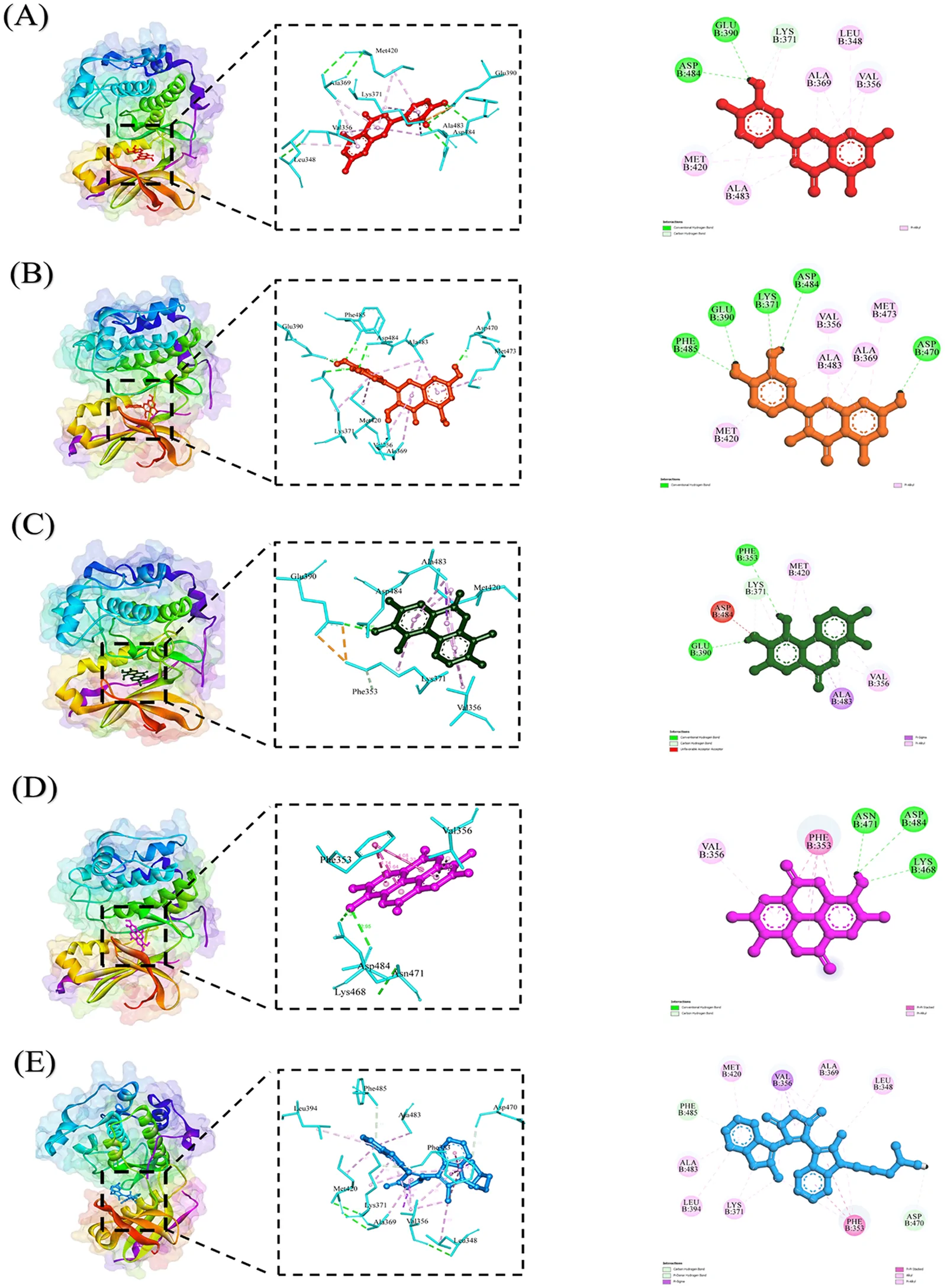
Comparative binding interactions of *T. chebula* phytoconstituents and control compounds with PRKCB catalytic site: (A) Luteolin, (B) Quercetin, (C) 3,4,8,9,10-pentahydroxydibenzo(b,d)pyran-6-one, (D) Ellagic Acid (control) and (E) Co-crystalized ligand 3-{1-[3-(Dimethylamino)propyl]-2-Methyl-1h-Indol-3-Yl}-4-(2-Methyl-1h-Indol-3-Yl)-1h-Pyrrole-2,5-Dione.

### 3.7. Machine learning-based QSAR modeling

The Random Forest regression model was developed to predict PRKCB inhibitory activity, focusing specifically on the top three phytoconstituents identified through molecular docking. The model achieved strong predictive performance, using an 80:20 training-to-validation split, with an R^2^ value of 0.91 and 0.81 for the training and test datasets, respectively, and RMSE of 0.41 and 0.58 for training and testing, respectively. These metrics indicate that the majority of experimental variability in pIC_50_ values was accurately reproduced. The predicted versus experimental scatter plot (Fig 9A) displayed a near-linear distribution, further confirming the reliability of the model. The Y-randomization analysis showed that the original model (R^2^ = 0.82) consistently outperformed all randomized models, confirming that its predictive performance was not due to chance correlation (Fig 9B). Furthermore, Permutation testing suggested that the original Random Forest model (R^2^ = 0.810) significantly outperformed all permuted models (p < 0.001), confirming its statistical robustness and predictive reliability (Fig 9C). Finally, Applicability domain analysis showed that 8 of 9 external test compounds (88.9%) were within the defined domain, indicating reliable predictions for the majority of structurally similar compounds (Fig 9D). Application of the optimized model to *T. chebula* phytoconstituents revealed clear trends in predicted inhibitory activity. Luteolin (predicted pIC_50_ = 6.661) and quercetin (predicted pIC_50_ = 6.312) emerged as the most active compounds, both exceeding the threshold for significant inhibitory potential (pIC_50_ ≥ 6). Another phytoconstituent, 3,4,8,9,10-pentahydroxydibenzo(b,d)pyran-6-one (predicted pIC_50_ = 5.859) (Table 4), displayed moderate activity, although slightly below the active cutoff. In contrast, Ellagic acid, included as a control compound, showed comparatively weaker inhibition (predicted pIC_50_ = 5.435). The consistent prediction of Ellagic acid as a weaker inhibitor provided an internal validation checkpoint, confirming that the model could differentiate compounds with varying potencies. Taken together, these findings confirm that the developed QSAR model meets international validation standards (R^2^ > 0.6; RMSE < 1.0) and offers mechanistic insight into the inhibitory potential of *T. chebula* phytochemicals. Importantly, the identification of luteolin and quercetin as strong candidates highlights their therapeutic relevance in DN through potential PRKCB inhibition.

**Table 4:** Random Forest QSAR Predicted pIC₅₀ Values of Key *T. chebula* Phytochemicals Against PRKCB.

Molecules Name	pIC_50_
Luteolin	6.661
Quercetin	6.312
3,4,8,9,10-Pentahydroxydibenzo(b,d)pyran-6-one	5.859
Ellagic Acid	5.435

*Note: pIC50 = Negative logarithm (-log*_*10*_ *) of the half-maximal inhibitory concentration (IC₅₀) measured in molar units.*

**Fig 9:**
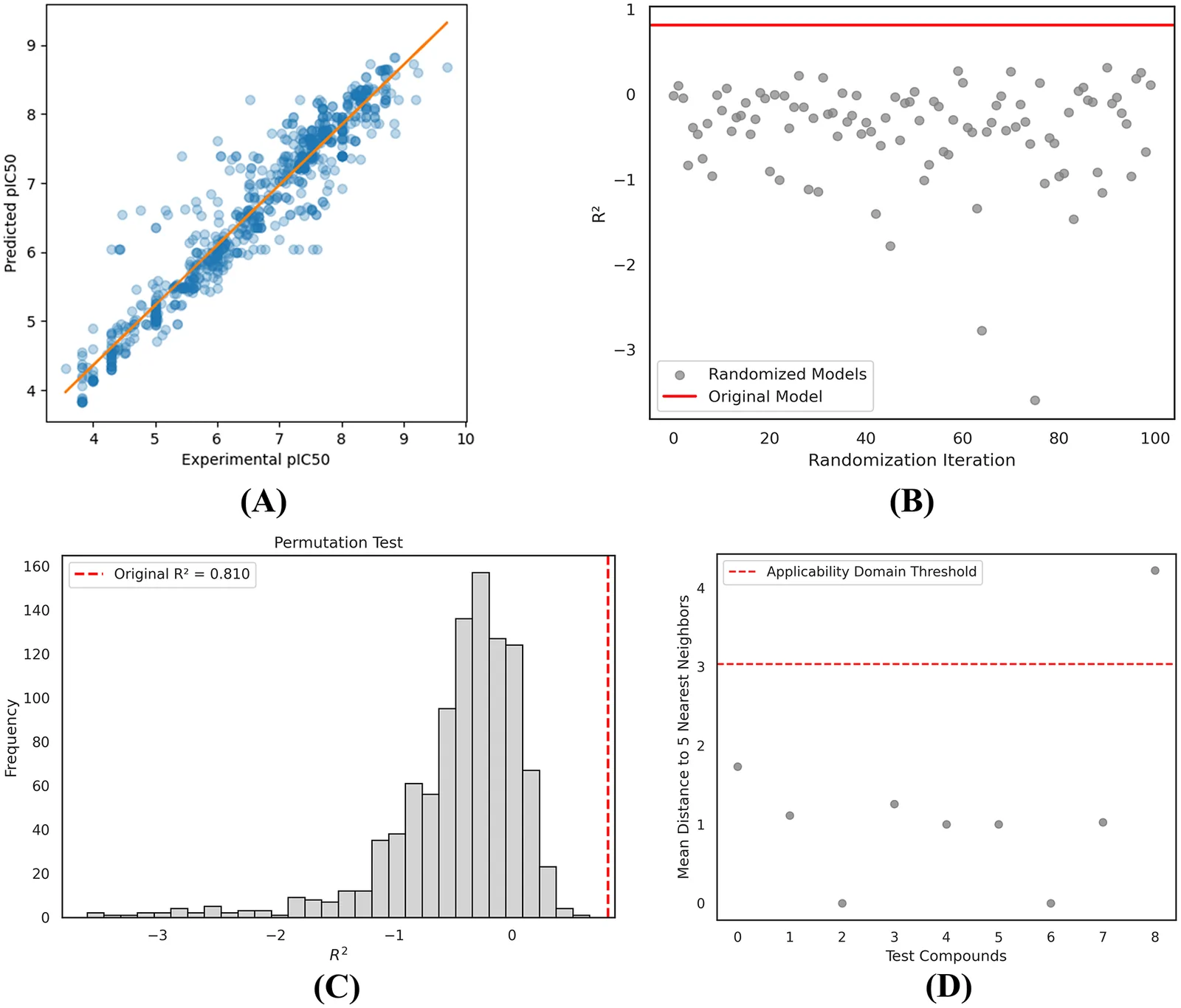
Machine Learning-Driven Validation of a Random Forest QSAR Model. (A) Correlating Predicted and Experimental pIC₅₀ Values for PRKCB Inhibition by *T. chebula* Phytoconstituents, (B) Y-randomisation, (C) Permutation testing, and (D) Applicability domain assessment of the testing dataset.

### 3.8. Quantum chemical insights

DFT calculations provide insights into quantum chemical properties to elucidate the electronic structure and intrinsic reactivity of the three selected bioactive ligands in comparison with the control, Ellagic Acid. All molecules were subjected to full geometry optimization at the B3LYP/6-31G(d,p) level of theory, followed by evaluation of frontier molecular orbitals (FMOs) and global reactivity descriptors. These parameters provide critical insights into the charge-transfer behavior, molecular stability, and chemical adaptability of small molecules toward biomolecular targets.

The orbital distribution analysis revealed that the HOMO densities of all ligands were predominantly localized over hydroxyl-bearing aromatic rings, emphasizing their capacity to act as electron donors. In contrast, the LUMO densities were concentrated around conjugated carbonyl systems, suggesting that these moieties serve as preferential electron-accepting sites during interactions with biological receptors. The quantitative reactivity descriptors showed clear variations among the compounds (Fig 10 and Table 5). Quercetin exhibited the narrowest HOMO-LUMO energy gap (0.14542 a.u.), the lowest hardness (0.07271 a.u.), and the highest softness (13.75326 a.u.), suggesting a greater tendency for charge transfer and electronic reactivity. Luteolin showed slightly higher values for the energy gap (0.15149 a.u.) and hardness (0.07574 a.u.), with a softness of 13.20219 a.u., indicating comparable electronic behavior. 3,4,8,9,10-Pentahydroxydibenzo(b,d)pyran-6-one was characterized by a gap of 0.16013 a.u., hardness of 0.08007 a.u., and softness of 12.48985 a.u., suggesting comparatively lower electronic reactivity. In contrast, Ellagic Acid displayed the broadest gap (0.19997 a.u.), the highest hardness (0.09999 a.u.), and the lowest softness (10.00150 a.u.), indicating reduced electronic responsiveness.

**Table 5:** Energy of HOMOs, LUMOs, gaps, hardness, and softness of selected compounds and the control compound.

Compounds	ɛHOMO	ɛLUMO	Gap	Hardness (Gap/2)	Softness, (1/Hardness)
Luteolin	-0.21453	-0.06304	0.15149	0.075745	13.20219
Quercetin	-0.20742	-0.062	0.14542	0.07271	13.75326
3,4,8,9,10-Pentahydroxydibenzo(b,d)pyran-6-one	-0.20209	-0.04196	0.16013	0.080065	12.48985
Ellagic Acid (Control)	-0.25454	-0.05457	0.19997	0.099985	10.0015

*Note: HOMO = Highest occupied molecular orbital; LUMO = Lowest unoccupied molecular orbital.*

**Fig 10:**
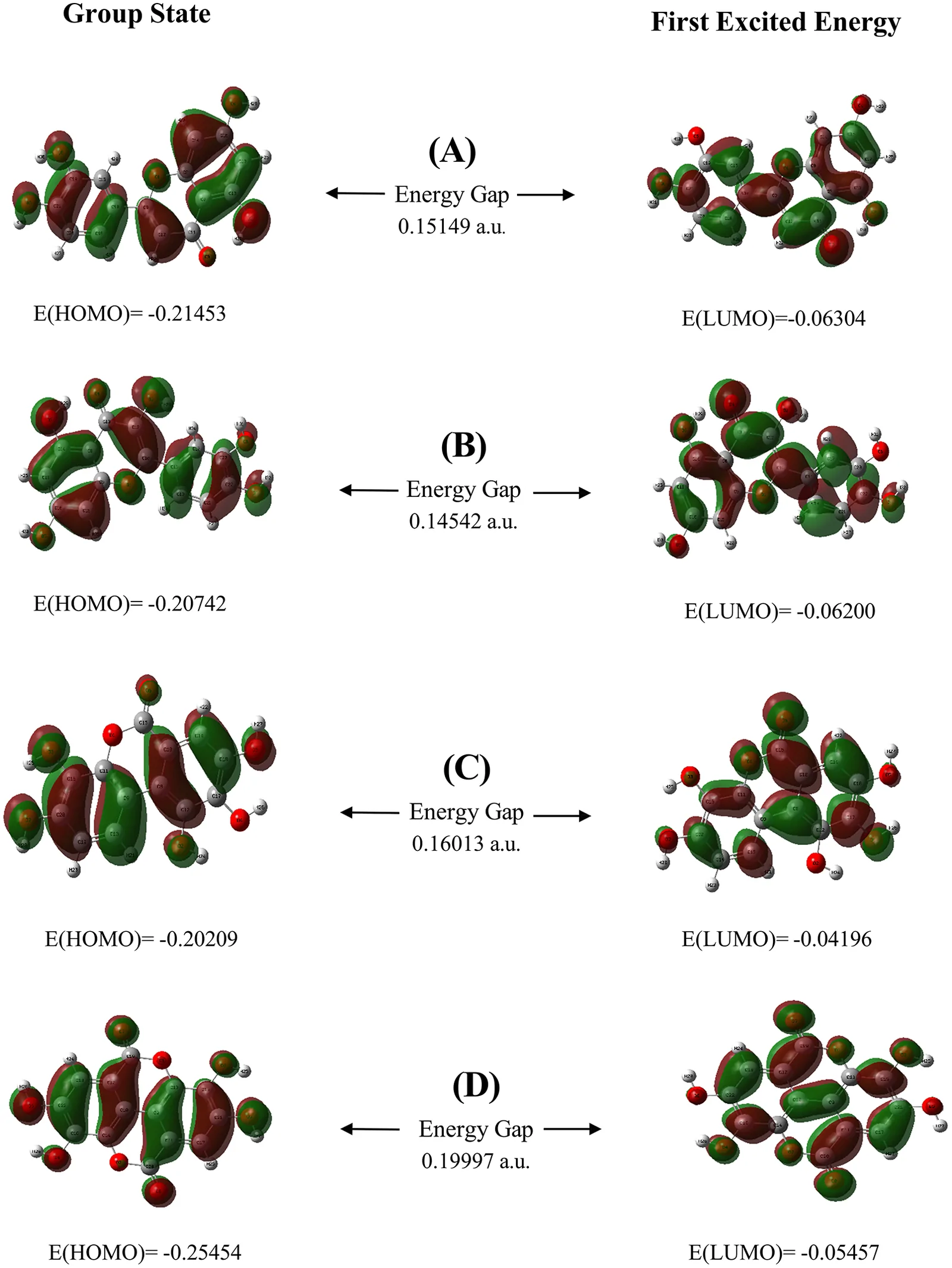
Comparative HOMO–LUMO distributions and reactivity parameters of bioactive ligands and control compounds. **(A)** Luteolin, **(B)** Quercetin, **(C)** 3,4,8,9,10-pentahydroxydibenzo(b,d)pyran-6-one, and **(D)** Ellagic Acid.

### 3.9. Molecular electrostatic potential (MEP) mapping

To complement the orbital-based findings, molecular electrostatic potential (MEP) analysis was performed to visualize the charge distribution and highlight the probable sites for electrophilic and nucleophilic interactions. MEP maps provide a three-dimensional representation of electron-rich and electron-deficient regions, thus predicting interaction preferences with biological macromolecules.

The analysis revealed that Luteolin and Quercetin displayed intense negative electrostatic potentials (red regions) localized around their hydroxyl and carbonyl groups, designating these sites as favorable for electrophilic attack. In both molecules, positive potentials (blue regions) were mainly observed around hydrogen atoms, which could engage in nucleophilic interactions (**Figs 11A-B**). 3,4,8,9,10-Pentahydroxydibenzo(b,d)pyran-6-one exhibited extensive negative potential around its multiple hydroxyl substituents, suggesting a pronounced tendency for hydrogen-bond formation, which may enhance its binding affinity in polar environments (Fig 11C). Conversely, Ellagic Acid also showed negative potentials over hydroxyl and carbonyl sites, but its broader energy gap and higher hardness reduced its adaptability in electronic redistribution, thereby limiting its reactivity compared to the test ligands (Fig 11D).

**Fig 11:**
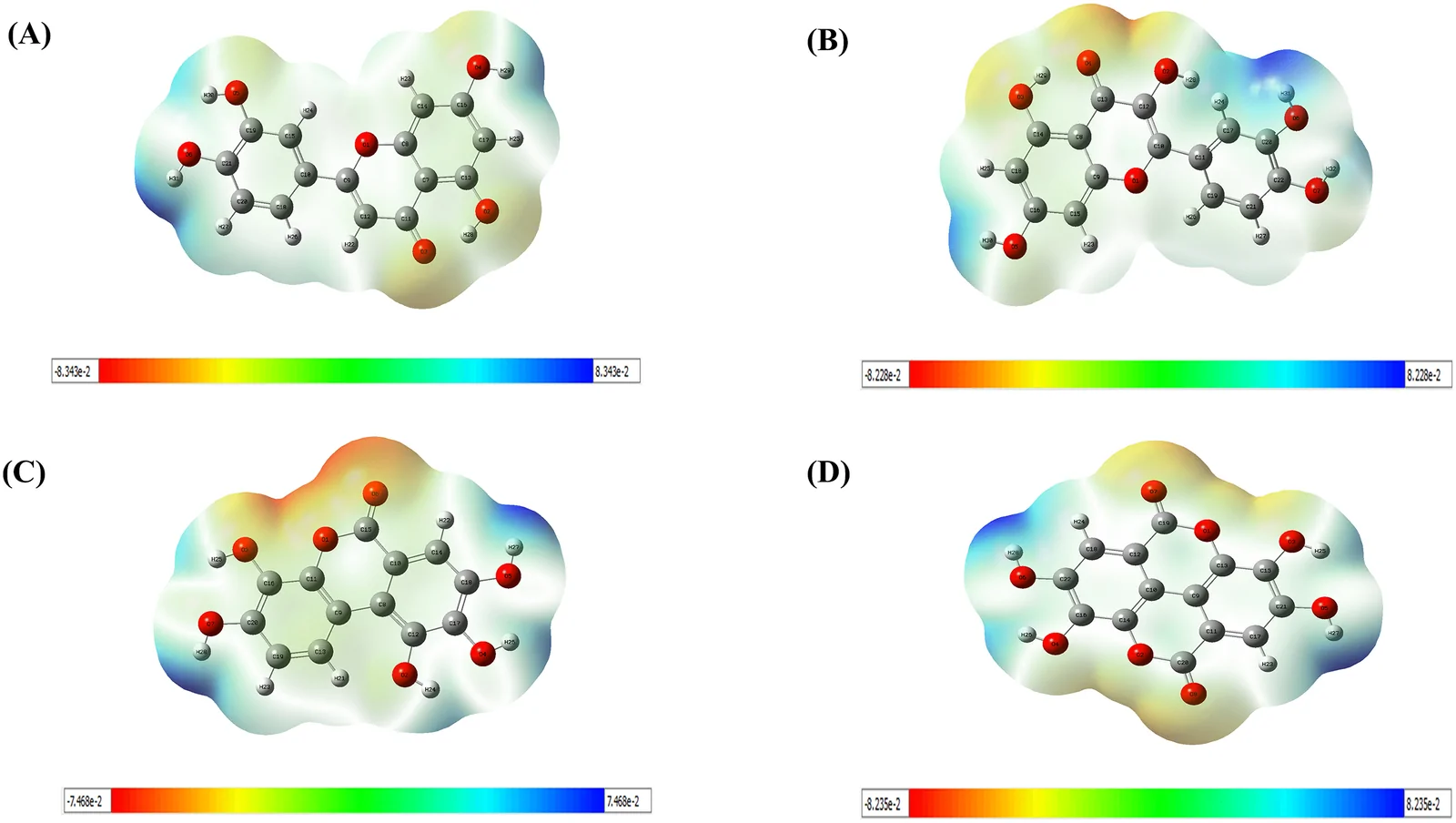
Molecular electrostatic potential (MEP) maps of *T. chebula* phytoconstituents and Ellagic Acid, showing electron-rich (negative) and electron-deficient (positive) regions: (A) Luteolin, (B) Quercetin, (C)3,4,8,9,10-Pentahydroxydibenzo(b,d)pyran-6-one, (D) Ellagic Acid.

### 3.10. MD simulation analysis

MD simulations were conducted to examine the structural stability, conformational flexibility, and dynamic characteristics of the protein-ligand complexes under conditions resembling the physiological environment. Unlike molecular docking, which provides only a fixed snapshot of the binding interactions, MD simulation provides a time-resolved depiction of molecular motion, revealing how the protein and ligand adapt to one another throughout the simulation period [107,108]. Key parameters, including the RMSD, RMSF, Rg, SASA, MolSA, PSA, and hydrogen bond (HB) profiles, were analyzed to assess the system’s structural behavior, stability, and binding persistence during the 100 ns simulation trajectory (**S5 Table**).

#### 3.10.1. RMSD, RMSF, and Rg analysis.

RMSD quantifies the average deviation of backbone atoms from the initial conformation and is a direct measure of the overall system stability. Small and steady RMSD values indicate that the protein-ligand complex has achieved equilibrium without major conformational shifts [109,110]. In the present study, all protein-ligand systems showed RMSD values within the acceptable range (1–4 Å), suggesting that the structures remained conformationally stable throughout the 100 ns simulation (Fig 12A) [111]. The average RMSD values for the protein backbone were 2.99 Å (18504424), 2.27 Å (5280343), 2.60 Å (5280445), and 2.36 Å (control complex). Among these, 5280343 displayed the lowest RMSD, indicating the highest structural stability and strongest conformational restraint within the binding pocket. The slightly higher RMSD in 18504424 reflects minor rearrangements at the loop regions but no major structural deviations. Overall, the smooth RMSD trajectories and stable plateaus across all complexes suggest that ligand binding did not disrupt protein stability.

**Fig 12:**
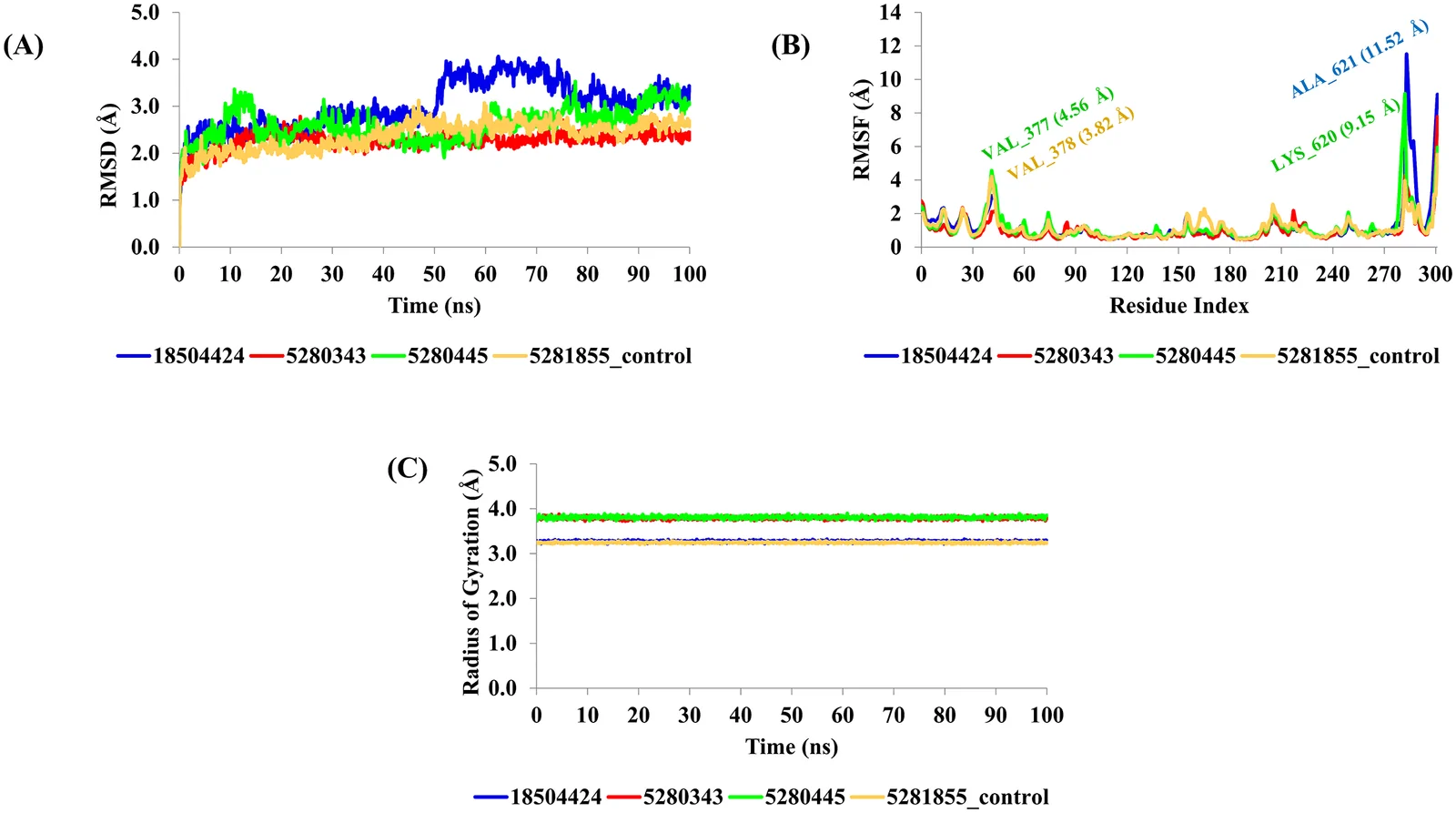
MD evaluation of PRKCB-ligand complexes. **(A)** RMSD shows stable backbone conformation, **(B)** RMSF highlights flexible regions of the protein, and **(C)** Rg confirms consistent structural compactness, indicating stable ligand-bound dynamics.

RMSF offers residue-specific insight into the flexibility of amino acids throughout the trajectory. Lower RMSF values suggest structural rigidity, while higher values typically correspond to flexible loops or terminal regions [112,113]. All complexes exhibited low RMSF values across most residues, indicating minimal flexibility and strong ligand-induced stabilization (Fig 12B). The mean RMSF values were 1.23 Å (18504424), 0.99 Å (5280343), 1.19 Å (5280445), and 1.12 Å (control). The highest fluctuations were observed at the N- and C-terminal ends, which are naturally more flexible. Among the tested compounds, 5280343 displayed the lowest overall RMSF, signifying a tighter interaction with the protein and reduced mobility of nearby residues. Furthermore, residue-wise RMSF analysis showed that VAL378 and VAL377 fluctuate moderately (3.82 Å and 4.56 Å, respectively), while LYS620 showed a higher fluctuation (9.15 Å), and ALA621 exhibited the highest fluctuation among all residues analyzed (11.52 Å), indicating this region as the most flexible part of the protein structure.

Rg measures the overall compactness and mass distribution of the protein throughout the simulation. Consistent Rg values suggest stable tertiary structure and compact folding, indicating that ligand binding does not cause unfolding or expansion [114,115]. The Rg profiles remained stable throughout the 100 ns trajectory for all complexes (Fig 12C). The mean Rg values were 3.27 Å (18504424), 3.79 Å (5280343), 3.81 Å (5280445), and 3.24 Å (control). The negligible variations (<0.1 Å) confirm that no major conformational rearrangements occurred during the simulation. These consistent Rg patterns suggest that the complexes retained their compact, globular configuration, supporting the notion of conformational equilibrium.

#### 3.10.2. Surface area parameters (SASA, MolSA, and PSA) and intermolecular interaction analysis.

SASA describes how much of the protein’s surface is exposed to solvent molecules, while MolSA and PSA represent the van der Waals and polar surface areas, respectively. Together, these parameters illustrate the interaction between the protein-ligand complex and the solvent environment, as well as the polarity of the binding interface [116,117]. The SASA values fluctuated moderately within the expected range, reflecting a balanced solvent interaction (Fig 13A). The mean SASA values were 205.96 Å² (18504424), 56.37 Å² (5280343), 129.99 Å² (5280445), and 125.77 Å² (control). The smaller SASA observed for 5280343 indicates deeper burial of the ligand within the protein pocket, suggesting tighter binding. Similarly, MolSA values remained nearly constant at 218.67 Å², 254.89 Å², 249.07 Å², and 227.13 Å² for the respective complexes (Fig 13B), implying no significant change in molecular surface topology. The PSA values ranged from 241.07 Å² (5280445) to 292.15 Å² (control), showing that all complexes maintained a balanced polar-nonpolar interaction surface (Fig 13C). The combination of reduced SASA and moderate PSA in 5280343 signifies strong hydrophobic and polar complementarity within the binding pocket.

**Fig 13:**
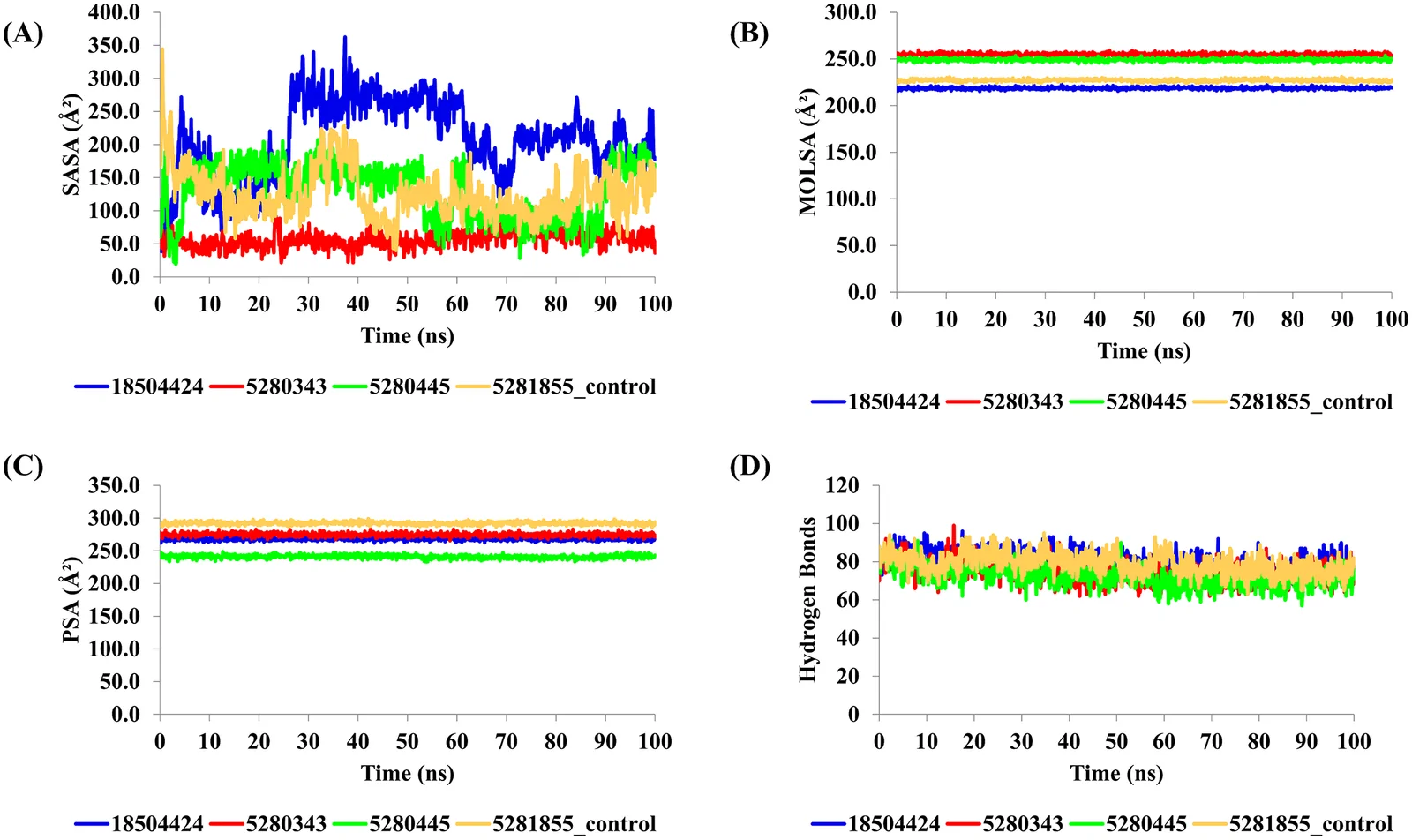
Surface area parameters: (A) SASA, (B) MolSA, (C) PSA of protein-ligand complexes, and (D) hydrogen bond analysis of protein-ligand complexes over a 100 ns simulation.

Furthermore, hydrogen bonds play a pivotal role in maintaining the stability of protein–ligand interactions. The number and persistence of these bonds throughout the trajectory serve as reliable indicators of binding strength and conformational stabilization [118–120]. Throughout the 100 ns trajectory, all systems maintained stable hydrogen bonding patterns (Fig 13D). The mean hydrogen bond counts were 80.27 (18504424), 74.62 (5280343), 72.35 (5280445), and 79.01 (control). Despite forming slightly fewer hydrogen bonds, 5280343 exhibited stronger persistence and uniform distribution, which likely contributed to its superior RMSD and Rg stability. Conversely, 18504424 displayed the highest number of hydrogen bonds, implying multiple interaction sites that reinforced its binding stability.

### 3.11. Post-simulation MM-GBSA binding free energy analysis

To better understand the energetic basis of ligand-protein interactions, post-simulation MM-GBSA calculations were carried out for the selected phytochemicals (CID: 5280343, CID: 5280445, and CID: 18504424) in comparison with the control drug. The binding free energy components, including van der Waals, Coulombic, solvation, hydrogen bonding, lipophilic, and packing contributions, were decomposed to provide a detailed view of the stabilizing forces governing complex formation (Fig 14).

**Fig 14:**
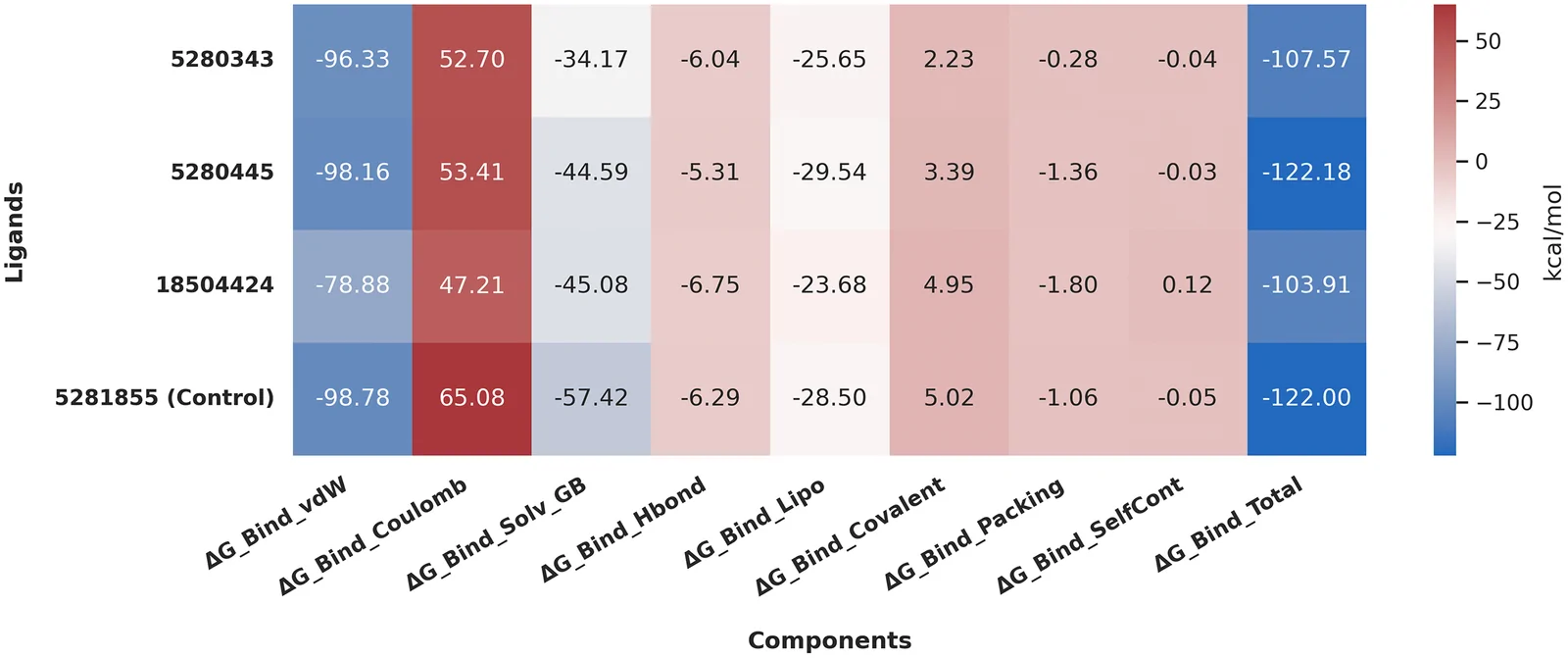
Post-simulation MM-GBSA binding free energy analysis of protein–ligand complexes.

The overall binding free energies indicated that all phytochemicals maintained favorable affinities comparable to the reference drug (ΔG_bind_total = −122.00 kcal/mol). Among the tested ligands, CID: 5280445 exhibited the strongest binding profile (ΔG_bind_total = –122.18 kcal/mol), nearly indistinguishable from the control, indicating its potential as a highly stable binder. CID: 5280343 also displayed a strong affinity (−107.57 kcal/mol), whereas CID: 18504424 showed a relatively weaker interaction (−103.91 kcal/mol), though still within a favorable range. In terms of energetic contributions, van der Waals interactions were the dominant stabilizing force across all ligands (−78.88 to −98.78 kcal/mol), underscoring the importance of hydrophobic contacts in maintaining stable complexes. Electrostatic interactions (Coulombic energy) further strengthened binding, especially for CID: 5280445 (53.41 kcal/mol) and the control (65.08 kcal/mol). By contrast, solvation free energy contributed unfavorably, yet this effect was offset by the dominance of van der Waals and lipophilic terms. Hydrogen bonding made modest stabilizing contributions (−5.31 to −6.75 kcal/mol), further supporting the binding stability.

## 4. Discussion

Network pharmacology integrated with advanced computational approaches has become an effective strategy for investigating the therapeutic potential of medicinal plants against complex diseases such as diabetic nephropathy (DN). In the present study, a comprehensive *in silico* framework was employed to explore the molecular mechanisms underlying the renoprotective effects of *T. chebula*. By integrating pharmacokinetic screening, target prediction, network pharmacology, molecular docking, machine learning-based QSAR analysis, density functional theory (DFT), molecular dynamics (MD) simulation, and MM/GBSA binding free energy analysis, the study identified the AGE-RAGE-PRKCB signaling axis as a key therapeutic target. Collectively, these findings suggest that *T. chebula* may reduce oxidative stress, inflammation, and extracellular matrix remodeling, which are major pathological processes involved in DN.

Pharmacokinetic and toxicity screening of 357 phytochemicals identified 14 compounds with favorable oral bioavailability, metabolic stability, and acceptable safety profiles. These compounds included flavonoids, terpenoids, and fatty acid derivatives, reflecting the chemical diversity of *T. chebula*. All selected compounds satisfied the Lipinski, Veber, and Ghose drug-likeness criteria, showed high gastrointestinal absorption, and exhibited no predicted mutagenic or hepatotoxic effects. These characteristics indicate their potential suitability for oral administration. Previous studies have also reported that *T. chebula* contains abundant polyphenolic compounds with antioxidant, anti-inflammatory, antidiabetic, and renoprotective properties.

To elucidate the molecular basis of *T. chebula* activity in DN, GO enrichment analysis was performed prior to PPI network construction. The 173 overlapping genes were significantly enriched in biological processes related to inflammation, oxidative stress, apoptosis, and extracellular matrix organization, all key features of DN. Cellular component analysis showed predominant localization in the extracellular space, plasma membrane, and exosomal compartments, suggesting that *T. chebula* may modulate inflammatory and fibrotic signaling at the cell-matrix interface. Molecular function analysis further revealed enrichment in cytokine activity, receptor binding, and integrin binding, highlighting their involvement in intercellular communication and signaling pathways associated with renal injury and repair [121–123].

Diabetic nephropathy (DN) often leads to end-stage renal disease. Oxidative stress demonstrates a crucial role in the onset and progression of DN, which triggers various pathological processes while promoting the activation of inflammation and forming a vicious oxidative stress-inflammation cycle that induces podocyte injury, extracellular matrix accumulation, glomerulosclerosis, epithelial-mesenchymal transition, renal tubular atrophy, and proteinuria. Conventional treatments for DN have limited efficacy. Polyphenols, as antioxidants, are widely used in DN with multiple targets and fewer adverse effects. This review reveals the oxidative stress and oxidative stress-associated inflammation in DN that lead to pathological damage to renal cells, including podocytes, endothelial cells, mesangial cells, and renal tubular epithelial cells. It demonstrates the potent antioxidant and anti-inflammatory properties by targeting Nrf2, SIRT1, HMGB1, NF-κB, and NLRP3 of polyphenols, including quercetin, resveratrol, curcumin, and phenolic acid. However, there remains a long way to a comprehensive understanding of the molecular mechanisms and applications for the clinical therapy of polyphenols. To explore their therapeutic potential at the molecular level, the 14 screened compounds were subjected to docking against the DN -associated PRKCB. The docking analysis revealed that three phytoconstituents, luteolin, quercetin, and 3,4,8,9,10-pentahydroxydibenzo(b,d)pyran-6-one, were the top-ranked compounds. Ellagic acid, a reported PRKCB inhibitor and an investigational bioactive compound with documented interactions involving PRKCB, was included as the reference ligand to provide a biologically relevant benchmark [104].

Previously reported PRKCB inhibitors were used to build a QSAR model, which was then applied to quantify the inhibitory potential of the top three ligands and examine their structure-activity relationships. Using bioactivity data derived from the ChEMBL database, a Random Forest regression model was trained and validated, yielding a high coefficient of determination (R^2^ = 0.91) and low RMSE (0.41), confirming the model’s robustness [124,125]. The predicted pIC₅₀ values reinforced the docking outcomes, with luteolin (pIC₅₀ = 6.66) and quercetin (pIC₅₀ = 6.31) emerging as the most potent inhibitors, followed by 3,4,8,9,10-pentahydroxydibenzo(b,d)pyran-6-one (pIC₅₀ = 5.86), surpassing the control compound ellagic acid (pIC₅₀ = 5.43) (Table 4). This finding is in agreement with their established antioxidant and signaling-modulatory properties, providing strong computational support for their potential as PRKCB modulators in DN.

DFT analysis was performed to evaluate the electronic properties of the selected phytochemicals. The calculated HOMO and LUMO distributions indicated favorable electron-donating and electron-accepting characteristics that may facilitate molecular recognition within the binding pocket of PRKCB. Quercetin displayed the smallest HOMO-LUMO energy gap and the highest softness, suggesting greater chemical reactivity and a higher ability to participate in intermolecular interactions. Luteolin exhibited comparable electronic behavior, whereas Ellagic acid exhibited a wider energy gap and lower adaptability, consistent with its weaker inhibitory profile. These descriptors provide complementary evidence supporting the observed binding behavior but should be interpreted as indicators of molecular reactivity rather than direct measures of biological potency. Molecular electrostatic potential (MEP) mapping highlighted hydroxyl and carbonyl groups as electron-rich sites, aligning with the residues identified in docking studies and confirming the complementarity between electronic properties and protein-ligand interactions [108,126].

Molecular dynamics simulations exhibited that ligand binding stabilized the PRKCB structure at both local and global levels. RMSF analysis revealed low residue fluctuations around the catalytic pocket, indicating rigid and stable ligand anchoring, with quercetin producing the greatest reduction in conformational flexibility.

Taken together, the findings of the present study suggest that *T. chebula* may exert renoprotective effects through a multi-target mechanism. Luteolin and quercetin, the primary bioactive flavonoids, exhibit favorable pharmacokinetics, high electron reactivity, and stable binding to PRKCB, a key isoform of protein kinase C. Mechanistically, hyperglycemia promotes the formation of advanced glycation end-products (AGEs), which interact with RAGE on renal cells, activating NADPH oxidase and generating reactive oxygen species (ROS). ROS in turn stimulate PRKCB via the phospholipase C (PLC) pathway, leading to MAPK/ERK activation and NF-κB nuclear translocation. This cascade upregulates pro-inflammatory and pro-fibrotic mediators, including VEGF, VCAM-1, TNF-α, interleukins, and RAGE, driving oxidative stress, inflammation, fibrosis, and renal tissue injury. By inhibiting PRKCB, luteolin and quercetin attenuate these downstream signaling events, thereby protecting renal tissues from hyperglycemia-induced damage (Fig 15). These results not only support previous experimental evidence of the antioxidant and renoprotective effects of *T. chebula* but also highlight PRKCB as a key molecular target in modulating the AGE–RAGE– PRKCB pathway.

**Fig 15:**
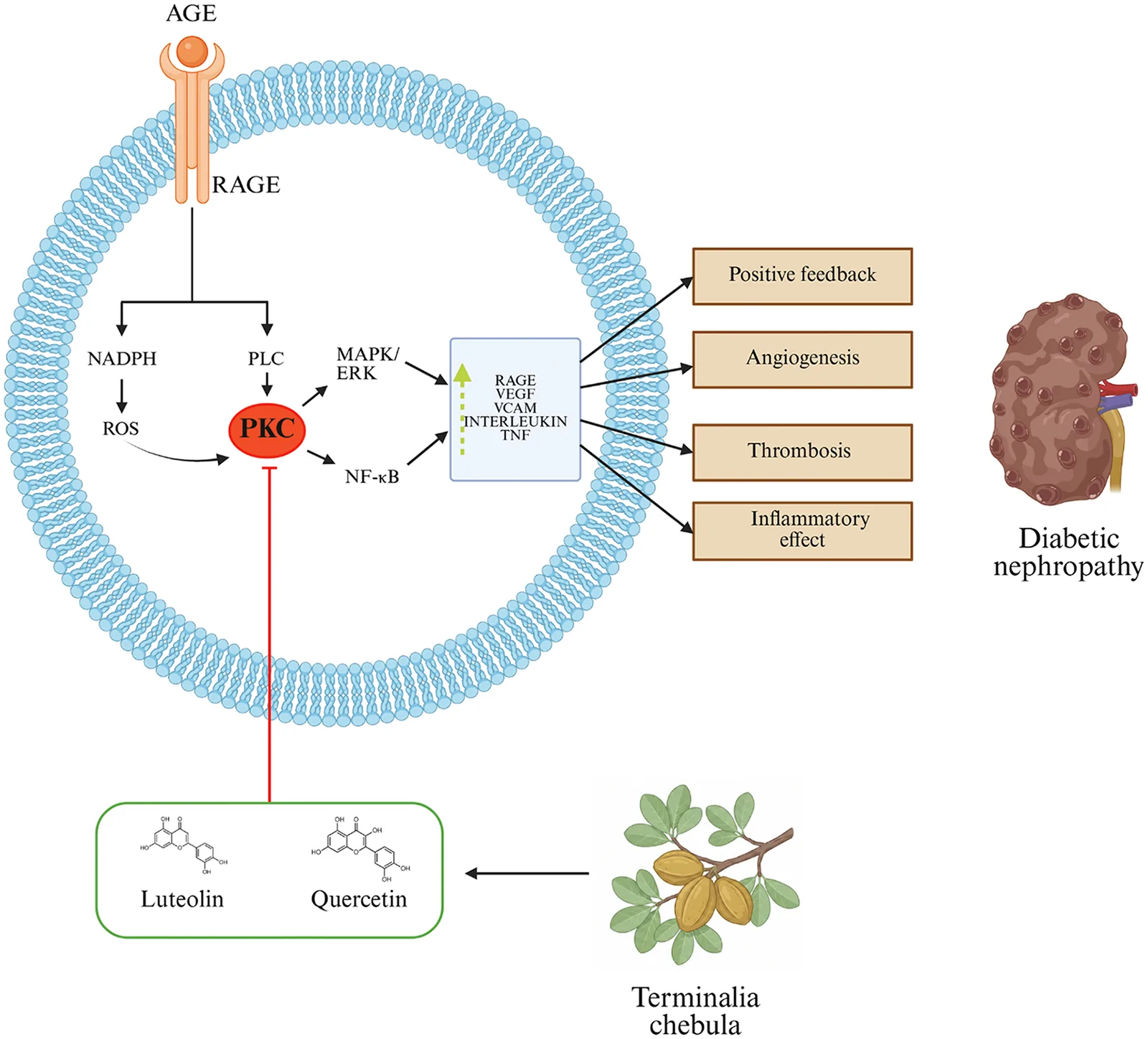
Proposed molecular mechanism illustrating the renoprotective action of luteolin and quercetin from *T. chebula* in DN (Designed using BioRender: https://BioRender.com/8jjquce).

Chronic hyperglycemia promotes the formation of AGEs, which interact with RAGE on renal cells and activate NADPH oxidase, resulting in excessive ROS production. Elevated ROS levels stimulate PRKCB via the PLC-dependent pathway, leading to activation of MAPK/ERK and NF-κB signaling [101]. This cascade enhances the expression of inflammatory and pro-fibrotic mediators such as VEGF, VCAM-1, TNF-α, interleukins, and RAGE itself, perpetuating oxidative damage, inflammation, and fibrosis within renal tissues. Luteolin and quercetin exhibit targeted inhibition of PRKCB, thereby interrupting this pathogenic signaling loop and mitigating renal injury associated with DN [127–131].

Overall, this study provides a comprehensive computational evaluation of the therapeutic potential of *T. chebula* against diabetic nephropathy. By integrating network pharmacology, molecular docking, QSAR modeling, DFT analysis, molecular dynamics simulation, and MM/GBSA binding free energy calculations, PRKCB was identified as a promising therapeutic target, while luteolin and quercetin emerged as the most promising bioactive compounds. These findings provide a strong theoretical basis for future experimental validation and support the continued development of *T. chebula*-derived phytochemicals as potential therapeutic agents for diabetic nephropathy.

## 5. Conclusion

This integrative computational investigation provides a mechanistic understanding of how *T. chebula* may exert renoprotective effects in DN. By uniting pharmacokinetic screening, differential gene expression integration, network pharmacology, molecular docking, machine learning-based QSAR modeling, quantum chemical analysis, MDS, and MM-GBSA energy estimation, the study delineates a coherent multi-target mode of action for this traditional medicinal plant. The findings reveal that the bioactive flavonoids luteolin and quercetin display favorable drug-likeness, electronic reactivity, and dynamic stability, enabling them to interact strongly withPRKCB, a pivotal hub in the AGE-RAGE signaling pathway. Through inhibition of PRKCB and concurrent modulation of key upregulated targets such as CCL2, COL1A1, FN1, CASP3, and EDN1, *T. chebula* phytochemicals may attenuate hyperglycemia-induced oxidative stress, inflammation, apoptosis, and extracellular matrix accumulation, all of which contribute to diabetic renal injury. Collectively, these results not only substantiate the traditional use of *T. chebula* as a renoprotective remedy but also establish a robust theoretical framework for its molecular pharmacology. The consistency of evidence across multiple computational dimensions underscores the reliability of these predictions and identifies *T. chebula*’s flavonoids as promising scaffolds against DN. However, further *in vitro* and *in vivo* investigations are necessary to validate these *in silico* insights, elucidate downstream molecular responses, and explore formulation or synergistic strategies that could translate *T. chebula*’s multi-target potential into clinically viable therapeutics for DN.

## Supporting information

S1 Table All the 357 compounds with their ADMET. (XLSX)

S2 Table Targets identification. (XLSX)

S3 Table GO and KEGG Pathways analysis using the DAVID database. (XLSX)

S4 Table Docking calculations of compounds against PRKCB. (XLSX)

S5 Table Molecular Dynamics simulation data analysis. (XLSX)
